# Deletion of Astrocytic TNFR1 Attenuates Hyperexcitability and Initiation of Epileptogenesis

**DOI:** 10.1002/glia.70205

**Published:** 2026-07-20

**Authors:** Lukas Henning, Ioannis Zalachoras, Giovanni Carriero, Gerald Seifert, Peter Bedner, Christian Steinhäuser, Andrea Volterra

**Affiliations:** ^1^ Institute of Cellular Neurosciences I University of Bonn, University Hospital Bonn Bonn Germany; ^2^ Department of Fundamental Neurosciences University of Lausanne Lausanne Switzerland; ^3^ Wyss Center for Bio and Neuroengineering Geneva Switzerland

**Keywords:** astrocyte, cytokine, gap junction channels, hippocampal sclerosis, status epilepticus, temporal lobe epilepsy, tumor necrosis factor alpha

## Abstract

Temporal lobe epilepsy (TLE), the most common form of adult focal epilepsy, is highly resistant to current medical therapy. Cytokine signaling, including via tumor necrosis factor‐alpha (TNFα), has been implicated not only as an accompanying factor but as a key aspect in the initiation of the disease. TNFα, through its type‐1 receptor (TNFR1) in astrocytes, controls excitatory circuits in the hippocampus, yet it remains unknown whether this astrocyte pathway specifically contributes to epilepsy. Here, we used a conditional cell‐specific knockout mouse line to induce TNFR1 deletion selectively in astrocytes and test its roles in the initiation and progression of TLE. Mice lacking astrocyte TNFR1 showed decreased basal spectral power, longer latency to first seizure following treatment with kainic acid, and a less severe phenotype up to 4 weeks later. Thus, abrogation of astrocyte TNFR1 signaling may be beneficial in TLE and could provide a new therapeutic target for this disease.

## Graphical abstract

- Inducible, astrocyte‐specific deletion of TNFR1 reduces basal EEG spectral power.
- Loss of astrocytic TNFR1 attenuates acute seizure activity in two distinct experimental models of epilepsy.
- Deletion of TNFR1 in astrocytes limits epilepsy development and hippocampal pathology in a mouse model of temporal lobe epilepsy with hippocampal sclerosis.
- Astrocytic TNFR1 mediates seizure‐induced loss of gap junctional coupling.

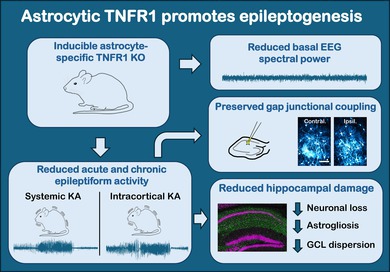

## 1 Introduction

Epilepsy is a neurological disease characterized by recurrent seizures caused by abnormal brain activity. It affects approximately 1% of the global population, making it one of the most common chronic neurological conditions (Fiest et al. 2017; Fisher et al. 2014). Temporal lobe epilepsy (TLE) is the most common form of adult focal epilepsy and is frequently refractory to medical therapy (Löscher et al. 2020). Accumulating evidence suggests that brain inflammation plays an important pathophysiological role in epilepsy. Inflammatory mediators released by brain cells during epileptic activity, such as cytokines, chemokines, danger signals, or prostaglandins, not merely drive local inflammation but also act as neuromodulators, influencing neuronal function and excitability (van Vliet et al. 2018; Vezzani and Viviani 2015). Furthermore, there is emerging evidence that their over‐expression is not only a consequence of epilepsy but a causal factor in the initiation of the disease in many cases (Eberhard et al. 2025; van Vliet et al. 2018; Vezzani et al. 2011; Vezzani and Viviani 2015). Of particular interest in this regard is the pro‐inflammatory cytokine TNFα, which exerts critical regulatory functions on synaptic circuits under normal conditions (Beattie et al. 2002; Santello et al. 2011; Santello and Volterra 2012; Stellwagen and Malenka 2006) and whose increased levels in pathology can lead to synaptic dysfunctions (Habbas et al. 2015). TNF‐α is initially produced as a transmembrane protein (tmTNFα), which is cleaved by the matrix metalloprotease TNFα‐converting enzyme (TACE, also known as ADAM17) at the cell surface, generating the soluble 17 kDa form of TNFα (sTNFα). The cytokine exerts its effects through two receptors, TNF receptor 1 (TNFR1 or p55TNFR) and TNF receptor 2 (TNFR2 or p75TNFR). TNFR1, which is widely expressed in most CNS cell types, is primarily activated by sTNFα. It contains a death domain that enables it to induce apoptosis or necroptosis under specific conditions. In contrast, TNFR2, which is mainly found on immune cells, is preferentially activated by tmTNFα. Unlike TNFR1, TNFR2 lacks a death domain and plays a role in promoting cell survival, tissue regeneration, and immune regulation (MacEwan 2002; Santello and Volterra 2012). The balance between TNFR1‐ and TNFR2‐mediated signaling, as well as the regulation of TNFα processing by TACE, are critical factors in determining the outcome of TNF‐α signaling and play a significant role in autoimmune disorders, cancer, or epilepsy.

In the case of epilepsy, several studies support the relevance of TNFα signaling in the development and progression of the disease (Balosso et al. 2005; Eberhard et al. 2025; Kirkman et al. 2010; Patel et al. 2017; Weinberg et al. 2013). In particular, elevated levels of TNFα have been reported to cause a number of astrocytic changes, such as reduced glutamate uptake, increased glutamatergic gliotransmission and disrupted inter‐astrocytic gap junctional (GJ) coupling, all of which may affect circuit excitability and potentially contribute to seizures (Bedner and Steinhäuser 2019; Habbas et al. 2015; Henning et al. 2023; Nikolic et al. 2018; Santello and Volterra 2012; Zou and Crews 2005). However, the exact role played by astrocyte TNFR1 in epilepsy has not been resolved. Therefore, we here developed and tested a mouse line with conditional astrocyte‐specific TNFR1 deletion and studied the specific contributions of this astrocytic receptor to changes in neuronal excitability and the initiation (i.e., the first hours after seizure onset) and progression of TLE.

## 2 Methods

### 2.1 Animals

**FIGURE 1:**
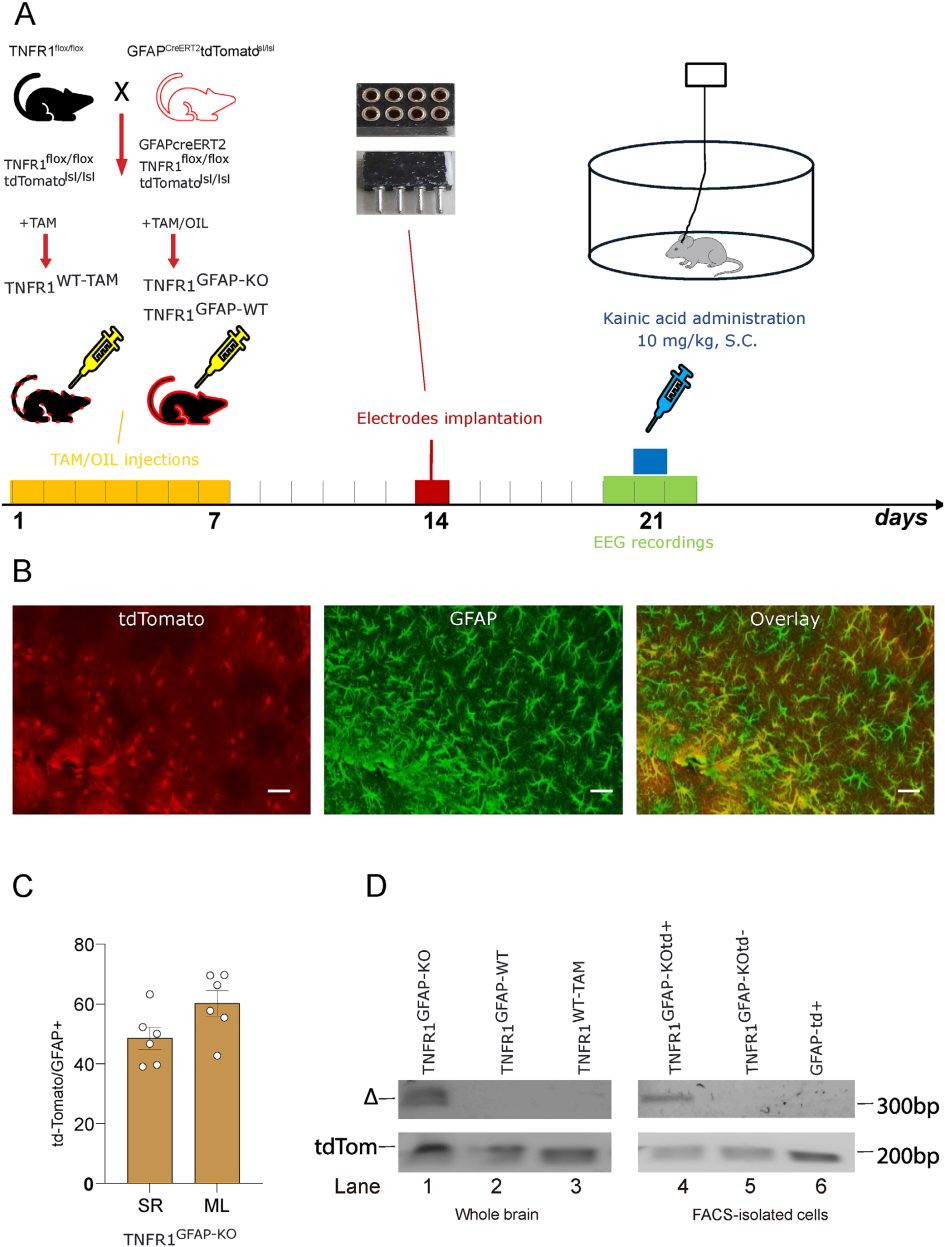
Experimental design and validation of the conditional astrocyte TNFR1 deletion mouse model. Schematic representing generation and validation of TNFR1^GFAP‐KO^ and related TNFR1^GFAP‐WT^ and TNFR1^WT‐TAM^ control mice, including experimental design and quantification of tdTomato expression in GFAP‐positive cells following TAM treatment. (A) *Top, left*: *TNFR1* ^*flox/flox*^ mice were crossed with *GFAP* ^*creERT2*^ *tdTomato* ^*lsl/lsl*^ mice to obtain *GFAP* ^*CreERT2*^ *TNFR1* ^*flox/flox*^ *tdTomato* ^*lsl/lsl*^ mice, amenable to TAM‐inducible *Cre*‐dependent recombination resulting in TNFR1 deletion and tdTomato expression in GFAP‐positive cells (TNFR1^GFAP‐KO^). This breeding scheme also resulted in the generation of *TNFR1* ^*flox/flox*^ *tdTomato* ^*lsl/lsl*^ mice. These mice were treated subcutaneously with 10 mg/kg TAM for 7 days (TNFR1^WT‐TAM^) to control for TAM‐induced effects unrelated to TNFR1 deletion. Together with *GFAP* ^*CreERT2*^ *TNFR1* ^*flox/flox*^ *tdTomato* ^*lsl/lsl*^ mice treated with an appropriate volume of vehicle (TNFR1^GFAP‐WT^, to control for *Cre* leakage), they formed the control group (Figure S1). *Top, middle*: A week after the last injection, mice stereotactically implanted with EEG electrodes bilaterally in the cortex and hippocampus were connected to an 8‐pin headmount (views from above and lateral). A grounding and a reference electrode were also implanted in groove holes above the cerebellum. *Top, right*: After a week of post‐operative recovery, mice connected to the EEG setup, were recorded under basal conditions for 24 h, then treated with Kainic acid (KA) (10 mg/kg, subcutaneously) and recorded for another 48 h. Seizures were monitored based on real‐time observation of the EEG signal mouse behavior. (B) TdTomato expression in GFAP‐positive cells in the CA1 hippocampus of TNFR1^GFAP‐KO^ mice. *Left*: tdTomato signal; *middle*: GFAP signal; *right*: Overlay. (C) Quantification of the number of tdTomato‐positive astrocytes in the hippocampus of TNFR1^GFAP‐KO^ mice. 48.47% ± 3.68% (mean ± SEM) of GFAP‐positive cells in the CA1 *stratum radiatum* (SR) and 60.24% ± 4.28% in the dentate molecular layer (ML) expressed tdTomato in mice sacrificed approximately 23 days following the beginning of TAM treatment. *N* = 6 mice. Scale bar = 40 μm. (D) Genomic PCR to validate TAM‐induced recombination and TNFR1 deletion. Δ denotes the expected PCR product following Cre‐dependent TNFR1 recombination. *Left*: Validation on whole brain tissue homogenates from TNFR1^GFAP‐KO^, TNFR1^GFAP‐WT^ and TNFR1^WT‐TAM^ mice (lanes 1–3). The expected product for the defloxed TNFR1 sequence (approximately 300 bp) is only present in TNFR1^GFAP‐KO^ mice, indicating lack of spontaneous recombination in the absence of *CreERT2* expression or TAM treatment. *Right*: Validation on FACS‐isolated astrocytes from the hippocampus and cortex of TNFR1^GFAP‐KO^ and *GFAP* ^*creERT2*^ *tdTomato* ^*lsl/lsl*^ mice (GFAP‐tdTom), where recombination only occurs in the tdTomato gene. The expected product for the defloxed TNFR1 sequence is only present in tdTomato‐positive cells isolated from TNFR1^GFAP‐KO^. It is, in contrast, absent in tdTomato‐negative cells from the same mice, or in tdTomato‐positive cells isolated from GFAP‐tdTom mice (lanes 4–6).

Mice carrying the tamoxifen‐inducible form of *Cre* recombinase (*CreERT2*) under the human glial fibrillary acidic protein (*hGFAP*) promoter (*GFAP* ^*creERT2*^ mice) were cross‐bred with a conditional tdTomato reporter mouse line (*tdTomato* ^*lsl/lsl*^), for two generations as previously described (Hirrlinger et al. 2006). The *GFAP* ^*creERT2*^ gene was maintained in heterozygosis. Subsequently, *GFAP* ^*creERT2*^ *tdTomato* ^*lsl/lsl*^ mice were crossed with *TNFR1* ^*fl/fl*^ mice, generously provided by Pr. George Kollias (Fleming Institute, Athens, Greece), to obtain *GFAP* ^*creERT2*^ *TNFR1* ^*flox/flox*^ *tdTomato* ^*lsl/lsl*^ and *TNFR1* ^*flox/flox*^ *tdTomato* ^*lsl/lsl*^ mice. These two latter lines, aged 90–120 days at the beginning of the experiments, were used for all experiments. Mice were housed two to five per cage in a 12 h:12 h light: dark cycle in a temperature‐ and humidity‐controlled environment. Food and water access were *ad libitum*. Both female and male mice were used in the study. In the *systemic* KA injection model (see below), recombination and TNFR1 deletion in astrocytes (TNFR1^GFAP‐KO^) were induced by treating *GFAP* ^*creERT2*^ *TNFR1* ^*flox/flox*^ *tdTomato* ^*lsl/lsl*^ mice intraperitoneally (i.p.) once daily for 7 days with tamoxifen (TAM, 100 mg/kg, Sigma‐Aldrich, Buchs, Switzerland) dissolved in corn oil (OIL, Sigma‐Aldrich) to a concentration of 10 mg/mL. This treatment has previously been shown to induce recombination in more than 30% of astrocytes in the hippocampus (Hirrlinger et al. 2006). In the *intracortical* KA injection model (see below), TAM preparation and treatment of the mice were performed as described (Henning et al. 2023). Briefly, for these experiments, mice were treated with 2 mg of tamoxifen daily for 5 days. In both procedures, similar recombination efficiencies were achieved (Figure 1C). *TNFR1* ^*flox/flox*^ *tdTomato* ^*lsl/lsl*^ littermates treated with TAM and *GFAP* ^*creERT2*^ *TNFR1* ^*flox/flox*^ *tdTomato* ^*lsl/lsl*^ mice treated with the appropriate volume of OIL were used as controls (TNFR1^WT‐TAM^ and TNFR1^GFAP‐WT^, respectively, collectively designated CTRL mice) (Figure S1A). Animal husbandry was performed according to EU and local Swiss and German governmental regulations. Experiments were approved by the Cantonal Veterinary Authorities in Vaud, Switzerland (authorization number: VD3115), the North Rhine–Westphalia State Agency for Nature, Environment and Consumer Protection, Germany (approval number: 81‐02.04.2020.A420) and carried out in accordance with the European Communities Council Directive of 22 September 2010 (2010/63/EU). We ensured that acute experiments were finished within 23 days following the first TAM injections, in order to maximize the number of astrocytes where recombination took place, while minimizing the potential synaptic contribution of maturing newborn granule cells deriving from neural precursors in the sub‐granular zone where recombination might have also occurred (Gu et al. 2012; Habbas et al. 2015).

### 2.2 Preparation of a Single‐Cell Suspension From Mouse Brain Regions, FACS Isolation of Astrocytes and Genomic PCR

A separate batch of TNFR1^GFAP‐KO^, TNFR1^WT‐TAM^ and TNFR1^GFAP‐WT^ mice were sacrificed with an overdose of pentobarbital (esconarcon; Streuli) and their brains were quickly removed and snap‐frozen in isopentane on dry ice and stored at −80°C until further processing. On the day of preparation, for each brain, a 1 mm‐thick coronal section including the cortex and hippocampus was taken to be used for FACS isolation of astrocytes, and another piece of brain tissue was taken for whole brain PCR. To FACS isolate astrocytes from frozen brain tissue, a protocol adapted from (Rubio et al. 2016) was used. Briefly, the section was placed in 1 mL of Hibernate‐A medium (Thermofisher) and the tissue was thoroughly minced with a razor blade in a glass Petri dish on ice. Afterwards, the solution including the minced tissue was transferred into a LoBind 1.5 mL Eppendorf tube (Eppendorf, Hamburg, Germany) and centrifuged at 110 g for 2 min at 4°C. The supernatant was discarded and 1 mL of ice cold Accutase solution (Merck) was added and gently mixed. Subsequently, samples were incubated for 30 min at 4°C with head‐over‐head mixing. Following enzymatic tissue digestion, samples were centrifuged at 960 g for 2 min at 4°C. After removing the supernatant, 0.6 mL of Hibernate‐A medium was added, and the samples were gently mixed. Samples were mechanically dissociated through four rounds of gentle trituration using 1.2, 0.8, 0.6 and 0.4 mm syringes. The cell suspensions were then centrifuged at 1700 g for 4 min at 4°C. After discarding the supernatant, the pellets were resuspended in Dulbecco's phosphate‐buffered saline (dPBS) containing 1% bovine serum albumin (BSA, Sigma‐Aldrich). Finally, samples were filtered through 100‐μm pore cell strainers and the flow through was collected for FACS sorting.

FACS analysis was performed on a BD FACS Chorus system, using a 100 μm nozzle, and conditions similar to those previously used (de Ceglia et al. 2023). Compensation was made on single‐color control for tdTomato, whereas gates were set on samples from TNFR1^GFAP‐WT^ mice (not expressing tdTomato). Forward scatter/side scatter gatings were used to remove clumps of cells and debris. For further analysis, 10,000 tdTomato‐positive and 10,000 tdTomato‐negative cells were sorted from TNFR1^GFAPKO^ mice and pelleted with centrifugation at 1700 g for 4 min at 4°C. Subsequently, DNA extraction was performed by adding 100 μL of extraction reagent (Quantabio, Beverly, MA, USA), mixed and incubated at 95°C for 30 min. After the incubation, 100 μL of stabilization buffer was added and samples were stored at –20°C until further processing. DNA extracts from whole brain samples were isolated in the same manner without prior FACS sorting.

PCR reactions were performed using the AccuStart II GelTrack PCR SuperMix (Quantbio), as per the manufacturer's instructions. To identify TNFR1 deletion, the following primers were used: *TNFR1Δ*: 300 bp. Forward: 5′‐CCTGCAGACACACGGGGAAA‐3′, reverse: 5′‐TGAACTCAGGTTGCCAGACG‐3′. The PCR conditions were: 95°C for 3 min, 32 cycles of 95°C for 45 s, 60°C for 40 s and 72°C for 1 min and 30 s, followed by an elongation step of 72°C for 10 min. To confirm the presence of the tdTomato gene, the following primers were used: *tdTomato* ^*lsl/lsl*^: 196 bp. Forward: 5′‐GGCATTAAAGCAGCGTATCC‐3′; Reverse: 5′‐CTGTTCCTGTACGGCATGG‐3′. The PCR conditions were: 95°C for 3 min, 35 cycles of 94°C for 30 s, 61°C for 30 s, and 72°C for 30 s, followed by an elongation step of 72°C for 5 min.

### 2.3 Systemic Kainic Acid (KA)‐Induced Acute Seizures Model

#### 2.3.1 Stereotactic Surgery for Electrode Implantation

Seven days after the last TAM or OIL injection, surgery for electrode implantation was performed, as previously described (de Ceglia et al. 2023). Briefly, mice were anesthetized by isoflurane inhalation in an induction chamber (isoflurane concentration of 2.5%) and maintained afterwards with an isoflurane concentration of 1.5%–2%. Mice were then mounted on a stereotactic frame (Kopf Instruments, Tujunga, CA, USA). Fur around the incision area was removed with depilation cream and the skin was sterilized with betadine. A mixture of lidocaine (Streuli, Uznach, Switzerland) (6 mg/kg) and carprofen (Rimadyl; Zoetis, Delémont, Switzerland) (5 mg/kg) was administered locally, 5 min before making a skin incision along the midline. Subsequently, the periosteum was removed to expose the skull. Six custom‐made coated stainless‐steel electrodes (AISI316LVM, bare diameter 0.125 mm, coated diameter 0.175 mm, Advent Research Materials, Oxford, UK) were implanted: two between the dura and the skull, bilaterally above the frontal cortex, two intrahippocampally (mediolateral: ±1.8 mm; anterior–posterior: −1.8 mm; dorsoventral: −1.9 mm), and two between the dura and the skull above the cerebellum, one as a reference and one as ground. Electrodes were fixed onto the skull with surgical glue and acrylic dental cement (Paladur, Kulzer, Hanau, Germany) and soldered to an EEG headmount (Pinnacle Technology, Lawrence, KS, USA) (Figure 1A). Mice were allowed to recover in their home cage for 7 days, while paracetamol (UPSA, Zug, Switzerland) was administered in their drinking water.

#### 2.3.2 Video and Electroencephalography (EEG) Recording Analysis of Acute Seizures Following Systemic KA

Following post‐operative recovery, mice were transferred to a plexiglas cage (Pinnacle Technology) and connected to the EEG recording system and allowed to acclimatize for 24 h, while EEG signal and video were recorded individually from each cage. On the following day, mice were injected subcutaneously with 10 mg/kg KA (Abcam, Watham, MA, USA) in sterile NaCl 0.9% (Bichsel, Unterseen, Switzerland). The EEG signal was continuously recorded at a sampling rate of 250 Hz for 48 h (Figure 1A). Detection of KA‐induced seizures was done in real‐time through inspection of EEG traces, videos and direct observation of the mice and later confirmed through manual scoring using the Sirenia seizure software (Pinnacle Technology). Analysis of seizures was performed throughout the 48 h recording period; however, seizures mostly occurred during the first 4 h following KA administration. Seizures were identified by visual inspection of synchronized EEG and video recordings and subsequently confirmed by manual offline analysis using Sirenia software. Seizures were defined as episodes of rhythmic epileptiform activity characterized by an abrupt onset of low‐amplitude high‐frequency discharges evolving into high‐amplitude rhythmic spike or spike–wave activity with a clear evolution in amplitude and frequency, lasting at least 15 s and easily distinguishable from baseline activity and movement‐related artifacts. Brief isolated spikes or interictal discharges were not classified as seizures. In addition, behavioral manifestations coinciding with EEG epileptiform activity were also taken into account according to the following criteria (Racine 1972): Stage‐1: absence‐like immobility; Stage‐2: hunching with facial or manual automatisms; Stage‐3: rearing with facial or manual automatisms and forelimb clonus; Stage‐4: repeated rearing with continuous forelimb clonus and falling; Stage‐5: generalized tonic clonic convulsions with lateral recumbence or jumping and wild running followed by generalized convulsions. Artifacts were removed manually. For each mouse, we scored and analyzed the latency between KA administration and the onset of the first seizure, the total number and duration of seizures and the average seizure duration.

Furthermore, the spectral EEG power was analyzed under basal conditions during the light phase of the first 24 h of EEG recordings, and also during KA‐evoked seizures, in the first 4 h following the drug administration, using LabVIEW 14.0 (National Instruments, Austin, TX, USA, application uploaded to Zenodo https://doi.org/10.5281/zenodo.17350183) and Labchart 8 (ADI instruments, Oxford, UK) software. Total spectral power was calculated, and Fast Fourier Transformation (FFT) was conducted to derive power values in different frequency bands, presented as area under the curve (AUC) values (Gnatkovsky et al. 2011). Post‐KA normalized total power values were calculated by dividing total spectral power following KA administration by total basal spectral power.

### 2.4 Intracortical KA‐Induced Chronic TLE‐HS Model

#### 2.4.1 KA Injection and Implantation of Telemetric EEG Transmitters

Unilateral intracortical KA injection as a model of TLE‐HS was performed as described previously (Bedner et al. 2015). Briefly, mice were anesthetized with a mixture of medetomidine (Cepetor, CP‐Pharma, Burgdorf, Germany, 0.3 mg/kg, i.p.) and ketamine (Ketamidor, WDT, Garbsen, Germany, 40 mg/kg, i.p.) and placed into a stereotaxic frame equipped with a manual microinjection unit (David Kopf; Tujunga, CA, USA). A total volume of 70 nL of a 20 mM solution of KA (Tocris, Bristol, UK) dissolved in 0.9% sterile NaCl was stereotaxically injected into the neocortex just above the right dorsal hippocampus. The stereotaxic coordinates were: 2 mm posterior to bregma, 1.5 mm from midline, and 1.7 mm from the skull surface. Immediately after KA injection, two holes were drilled 1 mm posterior to the injection site and 1.5 mm lateral from midline to insert two monopolar leads required for electrographic seizure detection. Telemetric transmitters (TA10EA‐F20 or TA11ETA‐F10; Data Sciences International (DSI), St. Paul, MN, USA) were implanted subcutaneously into the right abdominal region and the two monopolar leads were inserted 1 mm deep into the cortex. Attached leads were fixed to the skull using superglue and covered with dental cement (Paladur, Kulzer GmbH, Germany). The scalp incision was then sutured, and anesthesia stopped with atipamezol (Antisedan, Orion Pharma, Hamburg, Germany, 300 mg/kg, i.p.). To reduce pain, mice were injected with carprofen (Rimadyl, Pfizer, Karlsruhe, Germany) for 3 days. In addition, 0.25% Enrofloxacin (Baytril, Bayer, Leverkusen, Germany) was administered via drinking water to reduce the risk of infection. After surgery, mice were returned to clean cages and placed on individual radio receiving plates (RPC‐1; Data Sciences International, New Brighton, MN, USA), which capture data signals from the transmitter and send them to a computer running Ponemah software (Version 5.2, Data Sciences International). EEG recordings (24 h/day, for 4 weeks) were started directly after transmitter implantation and continued for 28 days after SE induction.

#### 2.4.2 Analysis of SE and Chronic Epileptiform EEG Activity in the TLE‐HS Model

EEG data were analyzed using NeuroScore (version 3.4.0) software (Data Sciences International) as described previously (Henning et al. 2023). Briefly, recordings were high pass filtered at 1 Hz and the number of seizures, their duration and spike numbers were determined using the spike train analysis tool implemented in NeuroScore based on the following criteria: threshold value = 7.5 × SD of the baseline (i.e., activity during artifact‐ and epileptiform‐free epochs 4 weeks after SE)—1 mV, spike duration = 0.1–50 ms, spike interval = 0.1–2.5 s, minimum train duration = 30 s, train join interval = 1 s, minimum number of spikes = 50. FFT was conducted to derive absolute *δ* (0–4 Hz), *θ* (4–8 Hz), *α* (8–12 Hz), *β* (12–30), and *γ* (30–50 Hz) power values (expressed in μV^2^), and the AUC (area under the curve) of normalized EEG power for individual frequency bands was calculated after normalization to baseline activity prior to conducting statistics. All animals included in the study developed SE, defined as > 5 min of continuous clinical and/or electrographic seizures or recurrent seizure activity without interim recovery. For SE quantification, the number and duration of seizures, as well as the total time spent in seizures, were assessed only during the first hour, since only within this period individual seizure durations could be accurately determined (Deshpande et al. 2020; Henning et al. 2023). In contrast, spike and spectral analyses were performed over the entire 4‐h recording period, which corresponds to the average duration of SE in this model (Bedner et al. 2015). The number of spontaneous generalized seizures during the chronic phase was determined manually from the EEG recordings by two experienced experimenters, according to the following criteria: rapid onset activity (typically in the delta and theta range) progressing towards rhythmic spiking and/or burst discharges with increasingly higher frequency components (typically 12–50 Hz, beta and gamma range), a clear deviation from baseline signal (at least > 2× baseline peak to peak amplitude; (Bedner et al. 2015; Pitsch et al. 2018)) and the occurrence of a postictal depression period.

### 2.5 Whole‐Cell Patch Clamp and Biocytin‐Filling of Astrocytes for Measuring Gap‐Junction Coupling in Hippocampal Slices

Four hours after intracortical KA injection, *GFAP* ^*creERT2*^ *TNFR1* ^*flox/flox*^ *tdTomato* ^*lsl/lsl*^ and *TNFR1* ^*flox/flox*^ *tdTomato* ^*lsl/lsl*^ mice were anesthetized with isoflurane (Piramal Healthcare, Morpeth, UK) and decapitated. Brains were quickly removed, and 200 μm‐thick coronal slices were cut on a vibratome (VT1000S, Leica Microsystems, Wetzlar, Germany) in ice cold preparation solution containing (in mM): 87 NaCl, 2.5 KCl, 1.25 NaH_2_PO_4_, 25 NaHCO_3_, 7 MgCl_2_, 0.5 CaCl_2_, 25 glucose, 75 sucrose, equilibrated with carbogen to stabilize pH (5% CO_2_/95% O_2_, pH 7.4). After storage of slices (15 min, 35°C) in preparation solution, slices were transferred to an artificial cerebral spinal fluid (aCSF) solution containing (in mM): 126 NaCl, 3 KCl, 2 MgSO_4_, 2 CaCl_2_, 10 glucose, 1.25 NaH_2_PO_4_, 26 NaHCO_3_, gassed with carbogen. To aid identification of astrocytes in the tissue, aCSF was supplemented with SR101 (1 μM, Sigma Aldrich, S7635, incubation 20 min, 35°C) (Kafitz et al. 2008). After SR101 staining, slices were transferred to aCSF and kept at room temperature (RT) for the duration of the experiments. For recordings, slices were transferred to a recording chamber and constantly perfused with aCSF. Patch pipettes fabricated from borosilicate capillaries with a resistance of 3–6 MΩ were filled with a solution containing (in mM): 130 K‐gluconate, 1 MgCl_2_, 3Na_2_‐ATP, 20 HEPES, 10 EGTA and biocytin (0.5%, Sigma Aldrich) (pH 7.2, 280–285 mOsm). To analyze gap junction coupling, SR101‐positive astrocytes were whole‐cell patch clamped and filled with biocytin for 20 min at RT. In addition to SR101 staining, astrocytes were identified by their characteristic morphology, passive current–voltage relationship, low input resistance and a resting membrane potential close to the Nernst potential of K^+^. Current signals were amplified (EPC 8, HEKA Electronic, Lambrecht, Germany), filtered at 3 or 10 kHz, and sampled at 10 or 30 kHz (holding potential −80 mV). Online analysis was performed with TIDA 5.25 acquisition and analysis software for Windows (HEKA) and Igor Pro 6.37 software (WaveMetrics, Lake Oswego, OR, USA). Voltages were corrected for liquid junction potentials. Only recordings matching the following criteria were included in the analysis: (i) resting potential negative to −60 mV, (ii) membrane resistance ≤ 10 MΩ, and (iii) series resistance ≤ 20 MΩ. After the recording, the pipette was carefully withdrawn and slices containing biocytin‐filled astrocytes were stored in 4% PFA‐containing PBS overnight at 4°C and subsequently transferred into PBS and stored at 4°C until immunohistochemistry. To label biocytin‐filled astrocytes in the tissue, slices were incubated in blocking solution with 2% Triton X‐100 and 10% normal goat serum (NGS) in PBS for 1 h and stained overnight with antibody solution containing rabbit anti‐GFAP (1:500, DAKO, Z0334, Hamburg, Germany). On the following day, slices were washed three times with PBS for 10 min each and subsequently incubated for 2 h at RT with anti‐rabbit Alexa Fluor 488 (1:500, A‐11034, Invitrogen, Karlsruhe, Germany) and streptavidin‐conjugated Alexa Fluor 647 (1:600, S21374, Invitrogen, Karlsruhe, Germany). Lastly, nuclear staining with Hoechst (1:200, diluted in dH_2_O) was performed (10 min, RT) followed by a final washing step (3× PBS, 5 min each) and slices mounted with Aquapolymount (Polysciences, Heidelberg, Germany) on objective slides. Astrocyte coupling efficiency was determined by manual counting of biocytin‐positive astrocytes in epileptic tissue of the various mouse groups unilaterally injected with KA, using the cell counter plugin implemented in FIJI. Cell counts of the ipsi‐ and contralateral hemispheres were calculated and compared between genotypes. Values exceeding mean ±2× SD were excluded as outliers prior to statistical analysis of GJ coupling data.

### 2.6 Tissue Preparation for Immunohistochemistry

For experiments involving immunohistochemistry, mice were euthanized with an i.p overdose of pentobarbital as indicated for the systemic KA experiments, or a solution containing 40 mg/kg ketamine (WDT) and 0.3 mg/kg, medetomidine (CP‐Pharma) for the intracortical KA experiments, administered i.p. and transcardially perfused with PBS, followed by perfusion with 4% paraformaldehyde (PFA) (Electron Microscopy Sciences, Hatfield, PA, USA) in PBS. Brains were harvested and post‐fixated overnight in 4% PFA‐containing PBS at 4°C. Some brains from chronically epileptic mice were post‐fixated for up to 48 h, depending on the quality of the initial fixation. Afterwards, PFA was removed, and brains were stored in new vials containing a fresh PBS‐sodium azide (0.05%) solution at 4°C. Coronal 40 or 50 μm‐thick sections were cut on a vibratome (Leica, Wetzlar, Germany) and stored in PBS‐sodium azide at 4°C until further processing.

### 2.7 Immunohistochemistry

Immunohistochemistry was performed using free‐floating slices and 24‐well plates in different experimental settings. In a subset of mice used in acute experiments, we quantified the number of astrocytes that were also tdTomato‐positive. For this, coronal 50 μm‐thick sections were cut on a vibratome (Leica, Wetzlar, Germany) and stored in PBS‐sodium azide at 4°C until further processing. Free‐floating sections were washed in PBS three times for 10 min, then blocked for 1 h in PBS‐0.5% Triton‐X (Sigma‐Aldrich) and 5% normal goat serum and subsequently incubated overnight with rabbit anti‐GFAP primary antibody (173,002, Synaptic systems, Göttingen, Germany) in a concentration of 1:500 at 4°C. Sections were then washed in PBS three times for 10 min and incubated for 3 h with a goat anti‐rabbit secondary antibody (Alexa 488, A‐11034, Thermofisher), at a concentration of 1:500 at room temperature (RT). Finally, sections were mounted with Fluoromount‐G mounting medium (Invitrogen, Waltham, MA, USA). To quantify tdTomato, the endogenous tdTomato fluorescence was detected.

For immunohistochemical staining of tissue from the chronic TLE‐HS mice, free‐floating slices from the dorsal hippocampus close to the KA injection site were used. For membrane permeabilization and blocking of unspecific epitopes, slices were incubated (1–2 h, RT) in 0.5% Triton X‐100 and 10% normal goat serum (NGS) in PBS. Slices were subsequently incubated overnight in primary antibody solution containing PBS on a shaker at 4°C. The following primary antibodies were used for staining: mouse anti‐NeuN 1:200 (Merck Millipore, MAB377, Darmstadt, Germany), rabbit anti‐GFAP 1:500 (DAKO, Z0334, Hamburg, Germany), rabbit anti‐Iba1 1:400 (Fujifilm Wako, #019‐19741, Neuss, Germany). Subsequently, sections were washed in PBS three times for 5 min and then incubated for 2 h with the appropriate secondary antibodies: goat anti‐rabbit 1:500 (Alexa Fluor488, A‐11034, Thermofisher), goat anti‐rabbit 1:500 (Alexa Fluor647, A‐21245, Invitrogen, Karlsruhe, Germany), streptavidin‐conjugated 1:300 (Cy3, S6402, Sigma Aldrich). To stain for NeuN in chronically epileptic tissue, slices were incubated with mouse anti‐NeuN primary antibody followed by goat anti‐mouse biotin (1:500, Dianova, AB_2338557, Hamburg, Germany) prior to incubation with streptavidin‐conjugated Cy3 antibody (1 h, RT). Next, slices were washed again three times with PBS (5–10 min) and nuclear staining was performed using Hoechst (10 min, RT). A final washing step (3× PBS, 5 min each) was performed and slices were mounted with Aquapolymount (Polysciences, Heidelberg, Germany) on objective slides and covered with cover slips. Slides were stored at 4°C before confocal imaging.

### 2.8 Confocal Microscopy and Image Analysis

For imaging of sections from acute systemic KA experiments, confocal images were captured using a Leica SP5 (Leica, Hamburg, Germany) confocal microscope. Z‐Stacks were acquired using a 20× objective (numerical aperture (NA) 0.75) and analyzed using FIJI (Free Software Foundation, Boston, MA, USA) software as previously described (Henning et al. 2023). Image resolution was 1024 × 1024 pixels at a speed of 400 Hz, with a pinhole size of 1 airy unit (AU) and z‐stacks were recorded at 0.5 μm. Laser excitation wavelengths were set at 488 nm for Alexa Fluor 488 and at 543 nm for tdTomato. Images were visualized using the LAS X software (Leica) (de Ceglia et al. 2023).

For imaging of sections from intracortical KA experiments confocal images were captured using a Leica SP8 microscope (Leica). Z‐Stacks were acquired using 10× (NA: 0.4), 20× (NA: 0.75) or 40× (NA: 1.1) objectives, and analyzed using FIJI (Free Software Foundation, Boston, MA, USA) or IMARIS8.0 (Bitplane, Zurich, Switzerland) software, as previously described (Henning et al. 2023). When performing imaging in chronically epileptic tissue, image resolution was set at 1024 × 1024 or 2048 × 2048 pixels recorded at a speed of 400 Hz and with a pinhole size of 1 AU. For gap‐junction coupling analysis, Hoechst, GFAP, and biocytin/AF647 signals were acquired using a 20× objective at a resolution of 1024 × 1024 pixels, with a scan speed of 400 Hz, a pinhole size of 1 AU, and a digital zoom factor of 1.2. Standard photomultiplier tubes were used for detection of fluorescent signals. Laser and detector settings were applied equally to all images acquired. Z‐Stacks were recorded at 0.5 μm (tdTomato), 2 μm (GJ coupling), 2.41 μm (Hippocampal sclerosis) or 0.42 μm (IBA1).

#### 2.8.1 Recombination Efficiency

The recombination efficiency was quantified by manually counting the fraction of GFAP‐positive cells that were co‐expressing tdTomato in the CA1 *stratum radiatum* (SR) and dentate molecular layer (ML) of the hippocampus using the cell counter plugin in FIJI software (Free Software foundation) in maximum intensity projections (MIPs) of z‐stacks.

#### 2.8.2 Hippocampal Sclerosis

Analysis of the extent of hippocampal sclerosis was performed as previously described (Deshpande et al. 2020; Henning et al. 2023, 2021). Briefly, granule cell dispersion (GCD), CA1 SR shrinkage, and the number of CA1 pyramidal neurons were estimated in MIPs. GCL width was measured at four positions indicated as T1–T4. T1 and T2 along a vertical line connecting the upper and lower cell layers of the DG, T3 and T4 at a distance halfway between the vertical line and the tip of the hilus. The average of the four values was used as an estimation of GCD. Shrinkage of the CA1 SR was determined by drawing a vertical line connecting the pyramidal and molecular layers, above the highest point of the dentate GCL. The length of the vertical line served as an indication of the remaining width of the SR. The degree of astrogliosis and microgliosis was estimated by quantifying the area occupied by the GFAP and IBA1 signals within individual regions of interest (ROIs) in the CA1 SR across all focal planes of each binarized image. The total volume occupied by the signals was subsequently derived by summing up the area calculated across all focal planes and was normalized to the total volume of the ROI prior to statistical analysis. GCD, SR width, and astrogliosis were quantified using FIJI software. The number of pyramidal neurons in the CA1 region was measured using the automated spot detection algorithm implemented in IMARIS8.0 within a 360 × 120 × 30 μm^3^ ROI located in the CA1 pyramidal layer just above the highest point of the dentate GCL.

### 2.9 Statistical Analysis

Statistical analyses and data visualization were performed either using R (R: A Language and Environment for Statistical Computing. Version 4.3.1. Vienna, Austria. 2023 Available from: https://www. R‐project.org/), OriginPro (OriginLab Corporation) or Graphpad prism software packages. For statistical comparisons between groups, two‐tailed independent samples *t*‐test, Welch's test or Mann–Whitney *U* test, Mantel‐Cox test, or two‐way ANOVA were used, as appropriate and indicated in the figure legends. Prior to statistical analyses of chronic KA experiments, data were checked for normality by inspection of histograms and Q–Q plots as well as by applying a Shapiro–Wilk test. In case of a non‐parametric data distribution, data were transformed according to Tukey's ladder of powers (Tukey, 1977) prior to performing two‐way ANOVA. Appropriate post hoc comparisons were performed to identify significant differences, that is in case of a significant interaction in the ANOVA (Independent samples *t*‐tests). Resulting *p*‐values were adjusted for multiple comparisons using the Benjamini–Hochberg method. *p*‐values < 0.05 were considered statistically significant. In case of a deviation from normality, data are presented as boxplots indicating the median and 25th and 75th percentiles, with whiskers showing the minima and maxima, or otherwise as mean ± standard error of the mean (SEM), as described in the figure legends. Grubb's outlier tests were performed in all data sets where KA was administered systemically, and significant outliers were excluded.

## 3 Results

### 3.1 Induction of Astrocyte TNFR1 Deletion and tdTomato Reporter Expression

To obtain mice with tamoxifen (TAM)‐inducible TNFR1 deletion in astrocytes, we crossed the *GFAP* ^*CreERT2*^ *tdTomato* ^*lsl/lsl*^ and *TNFR1* ^*flox/flox*^ mouse lines (Van Hauwermeiren et al. 2013; Hirrlinger et al. 2006), resulting in *GFAP* ^*creERT2*^ *TNFR1* ^*flox/flox*^ *tdTomato* ^*lsl/lsl*^ and *TNFR1* ^*flox/flox*^ *tdTomato* ^*lsl/lsl*^ mice (Figure 1A). *TNFR1* ^*flox/flox*^ *tdTomato* ^*lsl/lsl*^ littermates intraperitoneally treated with TAM (1 mg/kg, once per day for 7 days) (TNFR1^WT‐TAM^) and *GFAP* ^*creERT2*^ *TNFR1* ^*flox/flox*^ *tdTomato* ^*lsl/lsl*^ mice treated with corn oil (Sigma‐Aldrich, also once daily for 7 days) (vehicle, TNFR1^GFAP‐WT^) were used to control for Cre‐independent effects of TAM treatment and of genetic background, respectively. To confirm successful cell‐specific recombination in TAM‐treated *GFAP* ^*creERT2*^ *TNFR1* ^*flox/flox*^ *tdTomato* ^*lsl/lsl*^ mice (TNFR1^GFAP‐KO^), we used the astrocyte marker glial fibrillary acidic protein (GFAP) and counted the number of GFAP‐positive cells that also were tdTomato‐positive in two hippocampal regions, the CA1 SR and the dentate ML. Mice were sacrificed at the end of the experiment, approximately 23 days after the first TAM/OIL injection. This is a time point before GFAP‐positive neural precursor cells and newborn granule cells in the hippocampal dentate gyrus (DG) are fully mature and effectively incorporated into the hippocampal memory circuitry (Gu et al. 2012). Quantification of tdTomato‐positive cells indicated that 48.47% ± 3.68% (mean ± SEM) of all GFAP‐positive cells underwent TAM‐induced recombination in the CA1 SR and 60.24% ± 4.28% in the dentate ML (Figure 1B,C). Detailed previous analysis of lines containing the same *GFAP* ^*CreERT2*^ construct from the same lab and studied at similar age had shown analogous proportions of astrocytes undergoing recombination and virtually no neurons, oligodendrocytes, and microglia showing recombination (de Ceglia et al. 2023; Habbas et al. 2015). Here, we confirmed that tdTomato‐positive cells lacking concomitant GFAP expression were virtually non‐existent in CA1 SR and dentate ML, while the rate of recombination was similar to what has been previously observed (de Ceglia et al. 2023).

PCR analysis indicated lack of appreciable recombination in TNFR1^GFAP‐WT^ and TNFR1^WT‐TAM^ control mice, similar to what was previously observed (de Ceglia et al. 2023; Habbas et al. 2015). Thus, PCR of the genomic DNA from whole brain extracts highlighted the presence of the recombined (defloxed) TNFR1 gene in TNFR1^GFAP‐KO^ mice (Figure 1D, left side, lane 1 of the gel image), and, in parallel, lack of the defloxed TNFR1 product in TNFR1^GFAP‐WT^ and TNFR1^WT‐TAM^ controls (Figure 1D, left side, lanes 2 and 3, respectively), confirming that recombination occurred only in the presence of both Cre expression and TAM treatment. Validation in FACS‐isolated tdTomato‐positive astrocytes from TNFR1^GFAP‐KO^ animals confirmed the presence of the defloxed gene (Figure 1D, right side, lane 4), which was instead absent in tdTomato‐negative cells from the same mice and in tdTomato‐positive cells from a different line lacking the floxed TNFR1 sequence, that is *GFAP* ^*CreERT2*^ *tdTomato* ^*lsl/lsl*^ (GFAP‐td+) mice (Figure 1D, right side, lanes 5 and 6, respectively). The band for the genomic tdTomato gene was present in all samples and used as positive control for the presence of genomic DNA.

### 3.2 Astrocyte TNFR1 Deletion Attenuates Acute Seizures Induced by Subcutaneous Kainic Acid Injections

**FIGURE 2:**
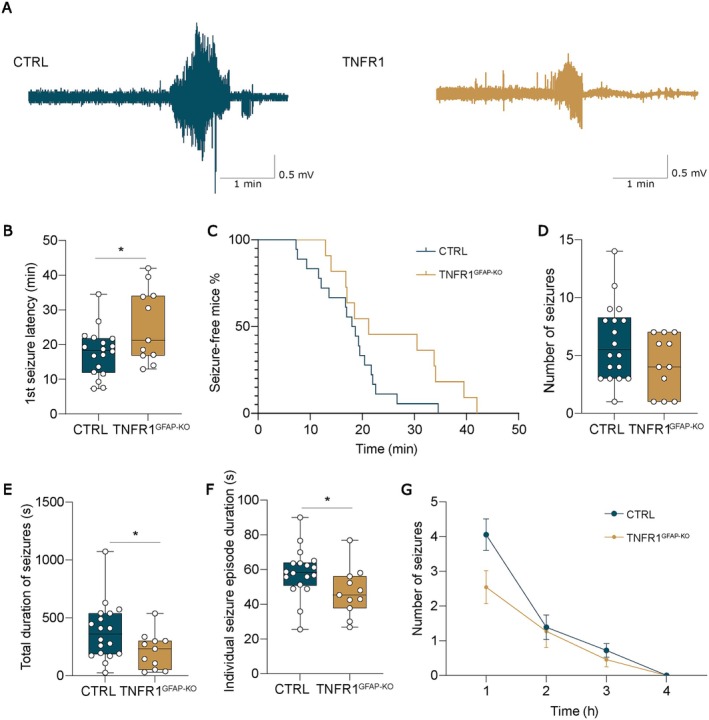
Reduced severity of KA‐induced acute seizures following astrocyte TNFR1 deletion. Protective effect of astrocyte TNFR1 deletion (TNFR1^GFAP‐KO^) against seizures induced by acute systemic KA administration. Having observed no difference between TNFR1^WT‐TAM^ and TNFR1^GFAP‐WT^ mice (Figure S1) and no recombination in these groups, we pooled them together for all analyses of ictal parameters (CTRL group). (A) Example EEG traces during seizures in CTRL (*left*) and TNFR1^GFAP‐KO^ mice (*right*). (B) Latency from KA administration to first seizure. The latency period was significantly longer in TNFR1^GFAP‐KO^ mice (brown) compared to CTRL mice (blue) (Welch's two‐tailed *t*‐test, *t*_15.15_ = 2.163, *p* = 0.047, *n* = 11–18 mice per group). (C) Proportion of mice remaining seizure‐free over time from KA administration. TNFR1^GFAP‐KO^ mice tended to remain seizure‐free longer than CTRL mice, but the effect did not reach statistical significance (Mantel‐Cox test, chi^2^ = 3.606, *p* = 0.058, *n* = 11–18 mice per group). (D) Total number of seizures observed up to 4 h following KA administration. The trend was as in B, but no significant difference was observed between groups (Welch's two‐tailed *t*‐test, *t*_25.77_ = 1.738, *p* = 0.094, *n* = 11–18 mice per group). (E) Total time that mice spent in seizures in the first 4 h following KA administration. Although the number of seizures was not different (panel C), their total duration was significantly lower in TNFR1^GFAP‐KO^ mice compared to CTRL mice (Welch's two‐tailed *t*‐test, *t*_26.91_ = 2.211, *p* = 0.036, *n* = 11–18 mice per group). (F) Duration of individual seizure episodes. Individual seizures were significantly shorter in TNFR1^GFAP‐KO^ mice compared to CTRL mice (Welch's two‐tailed *t*‐test, *t*_21.56_ = 2.092, *p* = 0.048, *n* = 11–18 mice per group). (G) Number of seizures in 1 h intervals over time. Two‐way ANOVA including the following terms: Group × time interaction (*F*_(3,81)_ = 2.621, *p* = 0.056), time (*F*_(2.135,57.64)_ = 44.25, *p* < 0.001) and group (*F*_(1,27)_ = 2.617, *p* = 0.1174), showed significant decrease in the number of seizures over time, but with no difference between groups. Data in panels A, C, D, and E are shown as boxplots with median and 25–75 percentiles, with whiskers indicating minimum‐maximum values. Panel C shows the proportion of seizure‐free mice at each time point for both groups. In panel F data are shown as mean ± SEM. *: *p* < 0.05.

To investigate the effects of astrocytic TNFR1 deletion on kainic acid (KA)‐induced acute seizures, 1 week after the last TAM/OIL treatment, mice were implanted with EEG electrodes in the cortex and hippocampus. One week following implantation, we connected the implanted mice (TNFR1^GFAP‐KO^, TNFR1^WT‐TAM^ and TNFR1^GFAP‐WT^) to the Pinnacle EEG setup for continuous EEG and video recording. Mice were allowed to acclimate under basal conditions for approximately 24 h before seizures were induced by subcutaneous systemic KA administration (10 mg/kg) (Figure 2A) (Rusina et al. 2021). We analyzed hippocampal EEG recordings made under both basal conditions and during the first 4 h following KA administration. Firstly, we saw no difference in any of the parameters analyzed between the two control groups in which TNFR1 expression was intact, TNFR1^WT‐TAM^ and TNFR1^GFAP‐WT^ (Figure S1A–F). Hence, we pooled these groups together for all subsequent statistical comparisons with TNFR1^GFAP‐KO^ mice. These comparisons showed that mice with astrocytic TNFR1 deletion displayed a delayed onset of the first KA‐induced seizure compared to control mice (Figure 2B), despite the two groups displaying a similar proportion of seizure‐free mice over time (Figure 2C) and a similar total number of seizures in the first 4 h following KA administration (Figure 2D). Moreover, seizure termination in TNFR1^GFAP‐KO^ mice was faster, resulting in significantly lower total seizure time (Figure 2E), as well as lower average seizure duration (Figure 2F) with respect to controls. Given the difference in seizure onset delay between groups, we also analyzed the number of seizures per hour for the first 4 h following KA administration, which was not significantly different between the two groups (Figure 2G). In conclusion, these data suggest that TNFR1 deletion in astrocytes delays the onset of KA‐induced seizures and reduces the duration of the seizure episodes, conferring partial resistance to the drug's epileptogenic action.

### 3.3 Astrocyte TNFR1 Deletion Reduces Basal EEG Spectral Power

**FIGURE 3:**
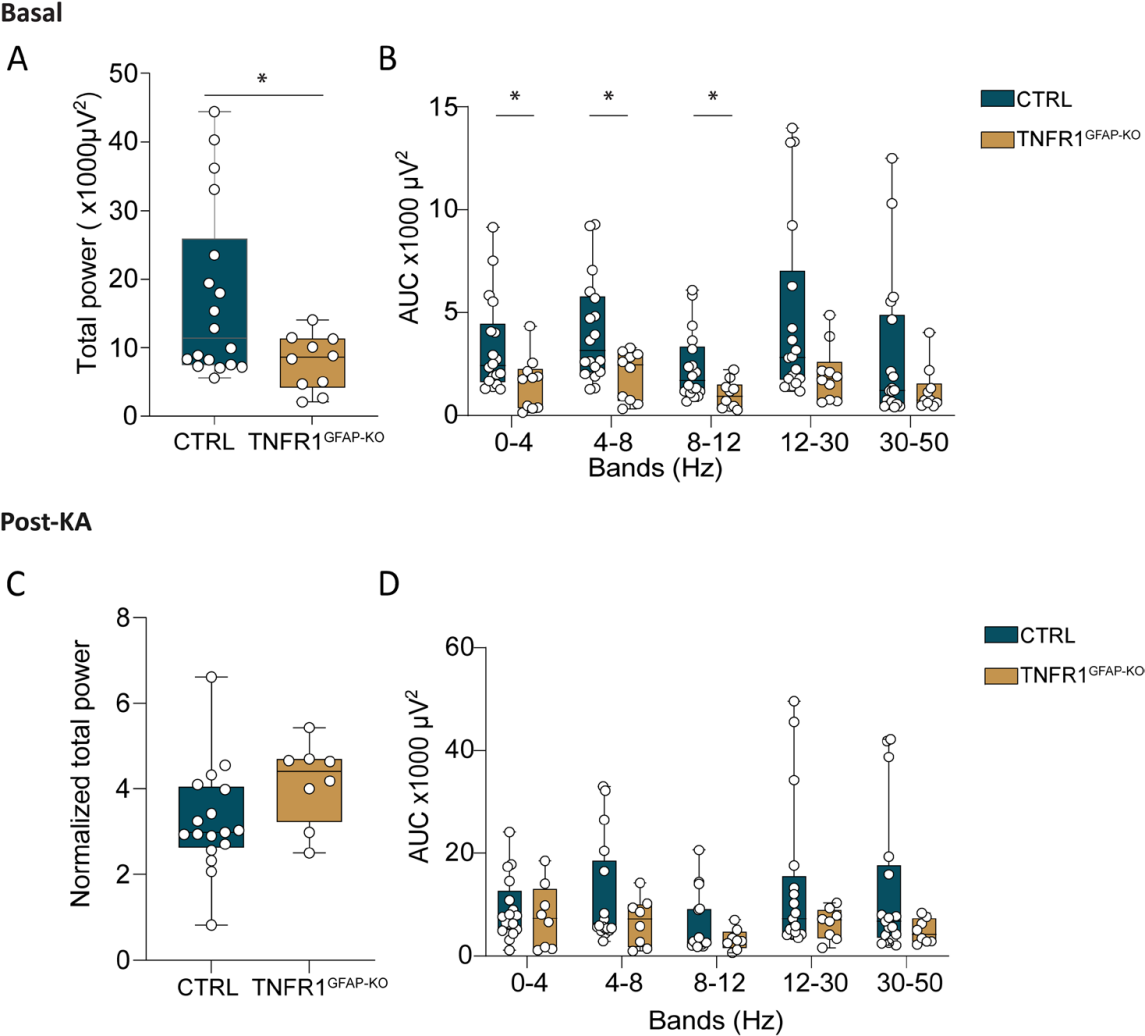
Astrocytic TNFR1 deletion diminishes basal spectral EEG power. Power spectra analysis in CTRL and TNFR1^GFAP‐KO^ mice under basal conditions (A–C) and in the first 4 h following systemic KA administration (D–F). Our data showed that TNFR1^GFAP‐KO^ mice had, under basal conditions, lower overall spectral EEG power than CTRL mice, an effect also seen at various frequency bands. Following KA administration, there was no difference between groups. (A) Baseline total EEG spectral power, shown as area under the curve (AUC) in ×1000 μV^2^. Total EEG spectral power was significantly lower in TNFR1^GFAP‐KO^ mice compared to CTRL (Mann–Whitney *U*‐test, *U* = 42, *p* = 0.021, *n* = 10–18 per group). (B) Baseline EEG spectral power in ×1000 μV^2^ in the different frequency bands. TNFR1^GFAP‐KO^ mice had lower power compared to CTRL in the lower frequency bands, but not in the bands above 30 Hz (Multiple Mann–Whitney tests between groups: *δ* = 0–4 Hz: *U* = 47, *p* = 0.04; *θ* = 4–8 Hz: *U* = 48, *p* = 0.045; *α* = 8–12 Hz: *U* = 39, *p* = 0.014; *β* = 12–30 Hz: *U* = 52, *p* = 0.072; *γ* = 30–50 Hz: *U* = 71, *p* = 0.382, *n* = 10–18 per group). (C) Total EEG spectral power following KA administration. In contrast to baseline recordings, normalized total power after KA was not different between TNFR1^GFAP‐KO^ and control mice (Welch's two‐tailed *t*‐test, *t*_(17.55)_ = 1.909, *p* = 0.073, *n* = 8–17 per group). (D) EEG spectral power in ×1000 μV^2^ in the different frequency bands following KA administration. EEG spectral power was not different between the two groups in any frequency band (Multiple Mann–Whitney tests: 0–4 Hz: *U* = 62, *p* = 0.754; 4–8 Hz: *U* = 57, *p* = 0.549; 8–12 Hz: *U* = 62, *p* = 0.754; 12–30 Hz: *U* = 49, *p* = 0.288; 30–50 Hz: *U* = 47, *p* = 0.238, *n* = 8–17 per group). In all panels, data are shown as boxplots with median and 25th‐75th percentiles, and whiskers indicating minimum‐maximum values. *: *p* < 0.05.

To further characterize the effects of astrocytic TNFR1 deletion in acute KA‐induced seizures, we performed spectral analyses of the EEG signal. First, we calculated the total spectral power as well as the power across the different frequency bands during the light phase of the EEG recordings under basal (KA‐naïve) conditions. Our data showed that, before KA administration, the total EEG spectral power, shown as area under the curve (AUC), in TNFR1^GFAP‐KO^ mice was significantly lower compared to control mice (Figure 3A). This effect in spectral power was visible across several individual bands (Figure 3B), including the *δ* (0–4 Hz), *θ* (4–8 Hz), and *α* (8–12 Hz) ranges.

We then calculated the total EEG power during the first 4 h following systemic KA administration. Our data indicate, in this case, that the normalized total spectral power (Figure 3C), as well as the power in each frequency band (Figure 3D), was not different between TNFR1^GFAP‐KO^ and control mice. Taking the effects on the EEG spectral power together with those on seizure onset and duration, TNFR1 deletion in astrocytes appears to cause a dampening of the overall level of circuit excitability, which may explain the delayed onset of seizures following systemic KA administration, as well as the shorter seizure duration compared to controls. Presumably, the circuit becomes less prone to enter into an epileptic state and more apt to exit it.

### 3.4 Astrocyte TNFR1 Deletion Attenuates the Severity of Status Epilepticus in a Mouse Model of Temporal Lobe Epilepsy With Hippocampal Sclerosis (TLE‐HS)

**FIGURE 4:**
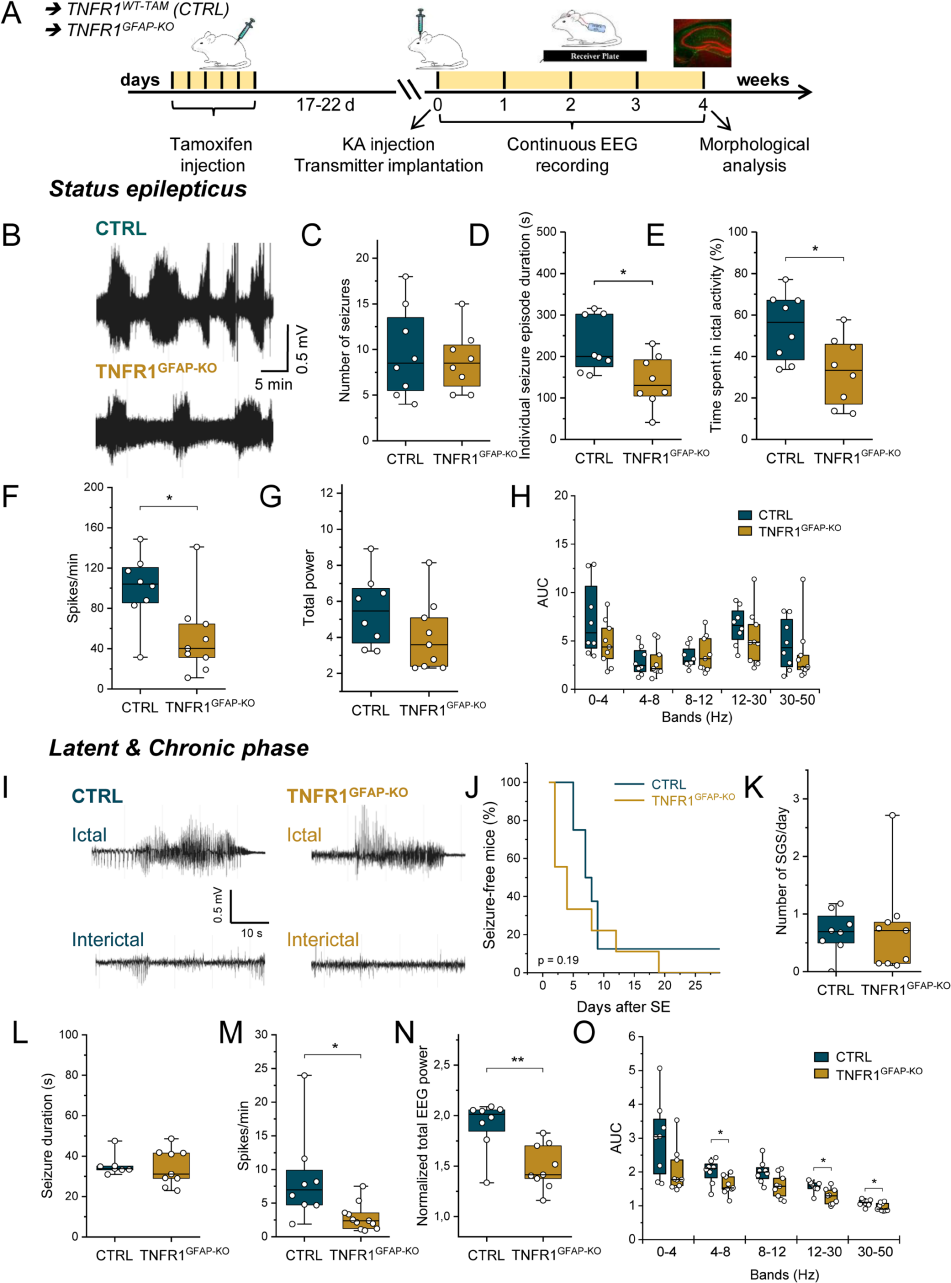
Astrocytic TNFR1 deletion attenuates the severity of KA‐induced acute and chronic epileptiform activity in a TLE‐HS model. (A) Schematic illustration of the experimental procedure. TNFR1^GFAP‐KO^ and TNFR1^WT‐TAM^ controls (CTRL) were i.p. injected with tamoxifen (2 mg/mouse/day) for 5 consecutive days. Seventeen to 21 days later, mice received stereotactic unilateral intracortical injections of KA, followed immediately by implantation of cortical electrodes and telemetry transmitters. The EEG was then recorded continuously over a period of 4 weeks. Mice were subsequently sacrificed, and the extent of histopathological hippocampal changes was assessed by immunohistochemical analysis. (B–H) Epileptiform activity in TNFR1^GFAP‐KO^ and TNFR1^WT‐TAM^ KA‐induced status epilepticus (SE). (B) Representative EEG traces during KA‐induced SE in CTRL (*top*) and TNFR1^GFAP‐KO^ (*bottom*) mice. (C) Number of seizures, (D) seizure duration, and (E) proportion of time spent in ictal activity during the first hour of SE. TNFR1 deletion reduced the duration (two‐tailed *t*‐test, *t*_14_ = 2.7, *p* = 0.017) and time spent in seizures (two‐tailed *t*‐test, *t*_14_ = 2.6, *p* = 0.021) but not the number of seizures (two‐tailed *t*‐test, *t*_14_ = 0.41, *p* = 0.68). (F) Number of spikes per min, (G) total (0.5–50 Hz) EEG power and (H) AUC (area under the curve) of normalized EEG power for individual frequency bands. TNFR1^GFAP‐KO^ mice displayed a significantly reduced number of spikes per min (two‐tailed *t*‐test, *t*_15_ = 2.72, *p* = 0.015) compared to CTRL mice, whereas total EEG power (two‐tailed *t*‐test, *t*_15_ = 1.48, *p* = 0.16) and power of the individual frequency bands were not significantly different between genotypes (Multiple Mann–Whitney *U* tests: 0–4 Hz: *U* = 23, *p* = 0.22; 4–8 Hz: *U* = 34, *p* = 0.88; 8–12 Hz: *U* = 37, *p* = 0.96; 12–30 Hz: *U* = 24, *p* = 0.27; 30–50 Hz: *U* = 27, *p* = 0.41, *n* = 8 (CTRL) and 9 (TNFR1^GFAP‐KO^) mice). (I–O) Epileptiform activity in TNFR1^GFAP‐KO^ and CTRL mice during the chronic phase of KA‐induced TLE‐HS. (I) Representative EEG traces (ictal activity, *top*; interictal activity, *bottom*) recorded 4 weeks post SE in TNFR1^GFAP‐KO^ and CTRL mice. (J) Length of the latent phase after SE; (K) total number of spontaneous generalized seizures (SGS)/day during the chronic phase; (L) Average SGS duration; (M) spike frequency; (N) Normalized total EEG power; (O) AUC of normalized EEG power in the different frequency bands. TNFR1^GFAP‐KO^ did not differ from CTRL mice in the duration of the latent phase (log‐rank test, *p* = 0.19) and in the number (Mann–Whitney *U* test, *U* = 38.5, *p* = 0.85) and duration (Mann–Whitney *U* test, *U* = 37, *p* = 0.6) of SGS, but exhibited a significantly lower spike frequency (Mann–Whitney *U* test, *U* = 75, *p* = 0.01) and EEG power than controls, including total power and power in some frequency bands (Multiple Mann–Whitney *U* tests: Total EEG power, *U* = 64, *p* = 0.008; power in different frequency bands: 0–4 Hz: *U* = 54, *p* = 0.09; 4–8 Hz: *U* = 160, *p* = 0.023; 8–12 Hz: *U* = 56, *p* = 0.06; 12–30 Hz: *U* = 63, *p* = 0.01; 30–50 Hz: *U* = 59, *p* = 0.03, *n* = 8 (CTRL) and 9 (TNFR1^GFAP‐KO^) mice). Open circles represent individual data points; *p < 0.05; ** < 0.01.

The reduced seizure activity observed early after systemic KA injection prompted us to also investigate the consequences of astrocyte TNFR1 deletion during later stages of epileptogenesis. To this end, we subjected TNFR1^GFAP‐KO^ and TNFR1^WT‐TAM^ control mice to the unilateral intracortical KA injection model, which reliably reproduces key morphological and functional features of chronic human TLE‐HS (Bedner et al. 2015; Jefferys et al. 2016). Epileptiform activity during status epilepticus (SE, see Methods) and the chronic phase were monitored by continuous telemetric EEG recordings (24 h/day) for 4 weeks (Figure 4A). For quantification of SE, we examined the number and duration of seizures as well as the time spent in ictal activity during the first hour of recording (see Methods, Deshpande et al. 2020; Henning et al. 2023). Although seizure frequency was not different between groups, seizure duration and time spent in ictal activity were significantly lower in TNFR1^GFAP‐KO^ mice (Figure 4B–E). To obtain a measure of the severity of the entire SE (Bedner et al. 2015), spike and spectral analyses were performed over an extended period of 4 h after SE induction. Interestingly, mice lacking astrocytic TNFR1 displayed significantly lower spike frequency than controls (Figure 4F). However, spectral analysis did not reveal any difference between genotypes, neither in normalized total power nor in any of the individual frequency bands (Figure 4G,H), consistent with observations in the systemic KA model (Figure 3C,D).

### 3.5 Astrocyte TNFR1 Deletion Attenuates Chronic Epileptic Activity and HS Development

After an equally long latent period, all TNFR1^GFAP‐KO^ mice and 87.5% (7 of 8) CTRL mice developed chronic spontaneous generalized seizures (SGS) (Figure 4I,J). Although frequency and duration of SGS did not differ between genotypes (Figure 4K,L), overall epileptiform activity (ictal and interictal) was reduced by astrocytic TNFR1 deletion, as both spike frequency (Figure 4M) and EEG power (total power and power of all frequency bands) were significantly lower in TNFR1^GFAP‐KO^ mice (Figure 4N,O).

Taken together, data from both the intracortical and systemic KA model demonstrate reduced acute seizure activity in astrocytic TNFR1‐deficient mice upon KA administration (Figure 2). Moreover, data from our TLE HS model demonstrate that chronic epileptiform activity is similarly attenuated in these mice, suggesting that astrocytic TNFR1 modulates neuronal excitability and contributes to the development and progression of TLE.

**FIGURE 5:**
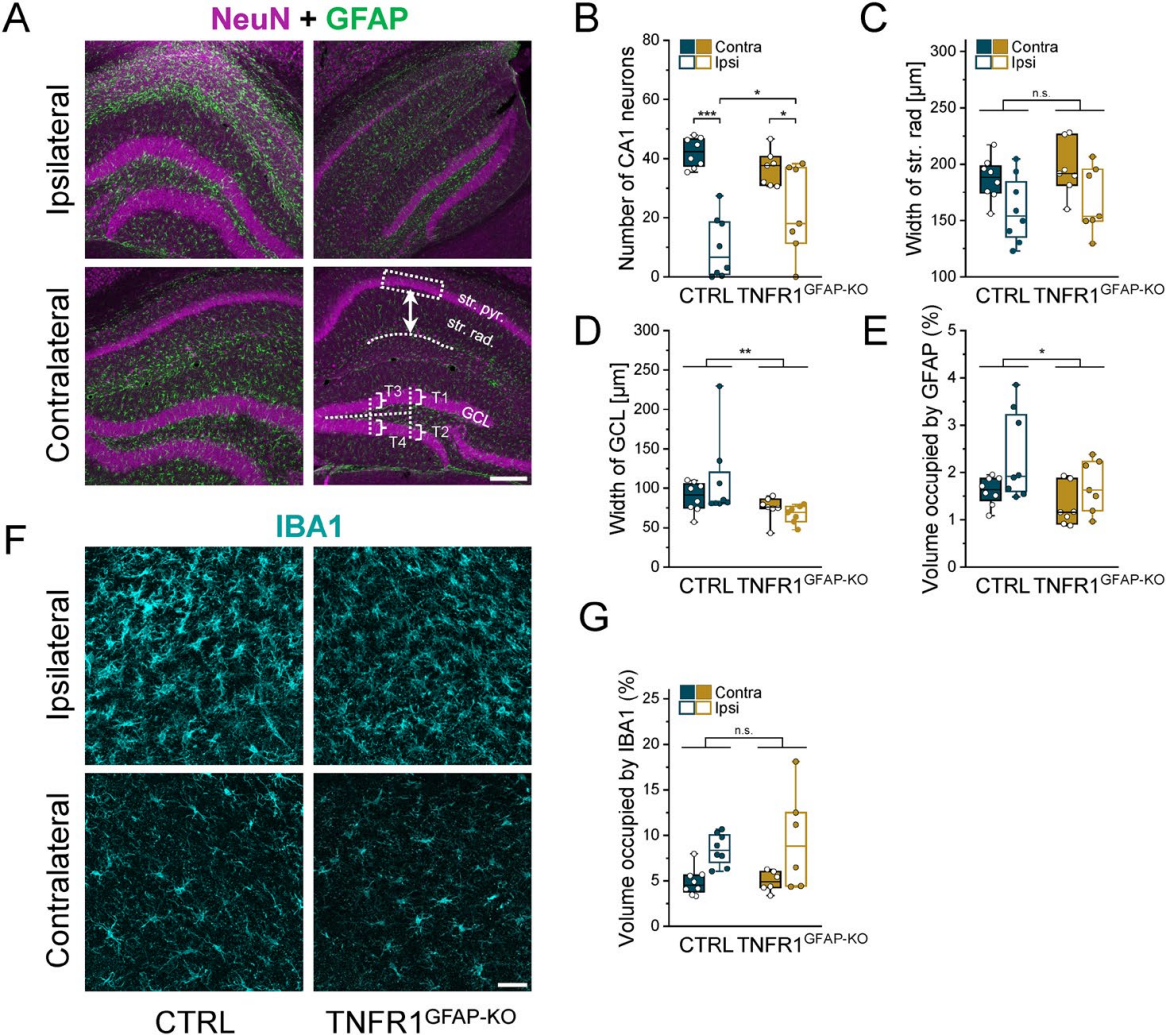
Astrocyte‐specific deletion of TNFR1 attenuates the extent of hippocampal sclerosis 4 weeks after intracortical KA injection. (A) Representative maximum intensity projections (MIPs) of combined NeuN (magenta) and GFAP (green) staining in ipsilateral (Ipsi) and contralateral (Contra) hippocampal slices from TNFR1^WT‐TAM^ (CTRL) and TNFR1^GFAP‐KO^ mice obtained 4 weeks after KA injection. Scale bar: 200 μm. Labeling in the lower right image: str. pyr.: *Stratum pyramidale*; str. rad.: *Stratum radiatum* (SR); GCL: Granule cell layer; T1–T4: Positions at which the GCL width was measured. The white box in str. pyr. indicates the ROI used for pyramidal neuron counting. The double‐headed arrow indicates the position at which the width of the SR was measured. (B–E) Morphological analyses of the extent of hippocampal sclerosis (HS) in CTRL and TNFR1^GFAP‐KO^ mice (*N* = 8 (CTRL) and 7 (TNFR1^GFAP‐KO^) mice; 3 slices/mouse). (B) Degeneration of ipsilateral pyramidal neurons was significantly less pronounced in mice lacking astrocyte TNFR1 than in CTRL mice (Two‐way ANOVA, significant effect of the site (*F* = 46.57_1,26_, *p* < 0.001) and significant interaction between side (Contra vs. Ipsi) and condition (CTRL vs. TNFR1^GFAP‐KO^) (*F* = 7.2_1,26_, *p* = 0.01). Results of the subsequent post hoc analysis of the interaction: Ipsi versus Contra (CTRL, *F* = 42.85_1,26_, *p* < 0.001; TNFR1^GFAP‐KO^, *F* = 7.51_1,26_, *p* = 0.01); CTRL versus TNFR1^GFAP‐KO^ (Ipsi, *F* = 5.97_1,26_, *p* = 0.043; Contra, *F* = 1.1_1,26_, *p* = 0.3)). (C) Shrinkage of the SR was not different between genotypes (Two‐way ANOVA: Significant effect of the site (*F* = 8.83_1,26_, *p* = 0.006) but not of the condition (*F* = 0.86_1,26_, *p* = 0.36) or the interaction of the two factors (condition and site, *F* = 0.001_1,26_, *p* = 0.97)). (D) Dispersion of the GCL was significantly less pronounced in TNFR1^GFAP‐KO^ than in CTRL mice (Two‐way ANOVA: Significant effect of the condition (*F* = 9.94_1,26_, *p* = 0.004) but not of the side (*F* = 0.02_1,26_, *p* = 0.88) or the interaction of the two factors (condition and site, *F* = 1.6_1,26_, *p* = 0.21)). (E) GFAP immunoreactivity, which reflects the extent of reactive astrogliosis, was significantly lower in TNFR1^GFAP‐KO^ than in CTRL mice (Two‐way ANOVA: Significant effect of the side (*F* = 7.04_1,26_, *p* = 0.01) and the condition (*F* = 6.01_1,26_, *p* = 0.02) but no of the interaction of the two factors (condition and site, *F* = 0.0003_1,26_, *p* = 0.98)). (F) Representative MIPs of IBA1 staining in the ipsi‐ and contralateral hippocampal CA1 SR from TNFR1^GFAP‐KO^ and CTRL mice 4 weeks after KA injection. Scale bar: 30 μm. (G) IBA1‐immunoreactivity did not differ significantly between genotypes (Two‐way ANOVA: Significant effect of the side (*F* = 17.05_1,24_, *p* < 0.001) but not of the condition (*F* = 0.0057_1,24_, *p* = 0.94) or the interaction of the two factors (condition and site, *F* = 0.15_1,24_, *p* = 0.7)). *N* = 8 (CTRL) and 6 (TNFR1^GFAP‐KO^) mice; 3 slices/mouse. Open circles represent individual data points; *p < 0.05; **p < 0.01; ***p < 0.001.

HS is a histopathological alteration of the hippocampus commonly seen in pharmacoresistant TLE patients and is characterized by prominent loss of pyramidal neurons in the CA1 and CA4 regions, reactive astro‐ and microgliosis, and granule cell dispersion (Blümcke et al. 1999). This histopathology is well replicated in our TLE‐HS model (Bedner et al. 2015). To examine the effects of astrocytic TNFR1 deletion on HS development, mice were sacrificed after 4 weeks of EEG recording and histopathological changes including neuronal cell loss and gliosis were assessed immunohistochemically (Figure 5). After KA injection, ipsilateral changes (neuronal loss, granule cell dispersion (GCD) and astrogliosis) were less pronounced in TNFR1^GFAP‐KO^ vs. control mice, while CA1 SR shrinkage and microglial reactivity remained unaffected by astrocytic TNFR1 deletion (Figure 5). Interestingly, the width of the granule cell layer (GCL) was reduced not only ipsilaterally but also contralaterally, suggesting that astrocytic TNFR1 may influence GCL architecture (Figure 5A,B).

Together, these data demonstrate less pronounced HS in mice lacking astrocytic TNFR1, indicating that TNFR1 signaling in astrocytes is implicated in the development of hippocampal pathology in TLE.

### 3.6 Astrocytic TNFR1 Mediates Seizure‐Induced Loss of Gap Junctional Coupling of Astrocytes

**FIGURE 6:**
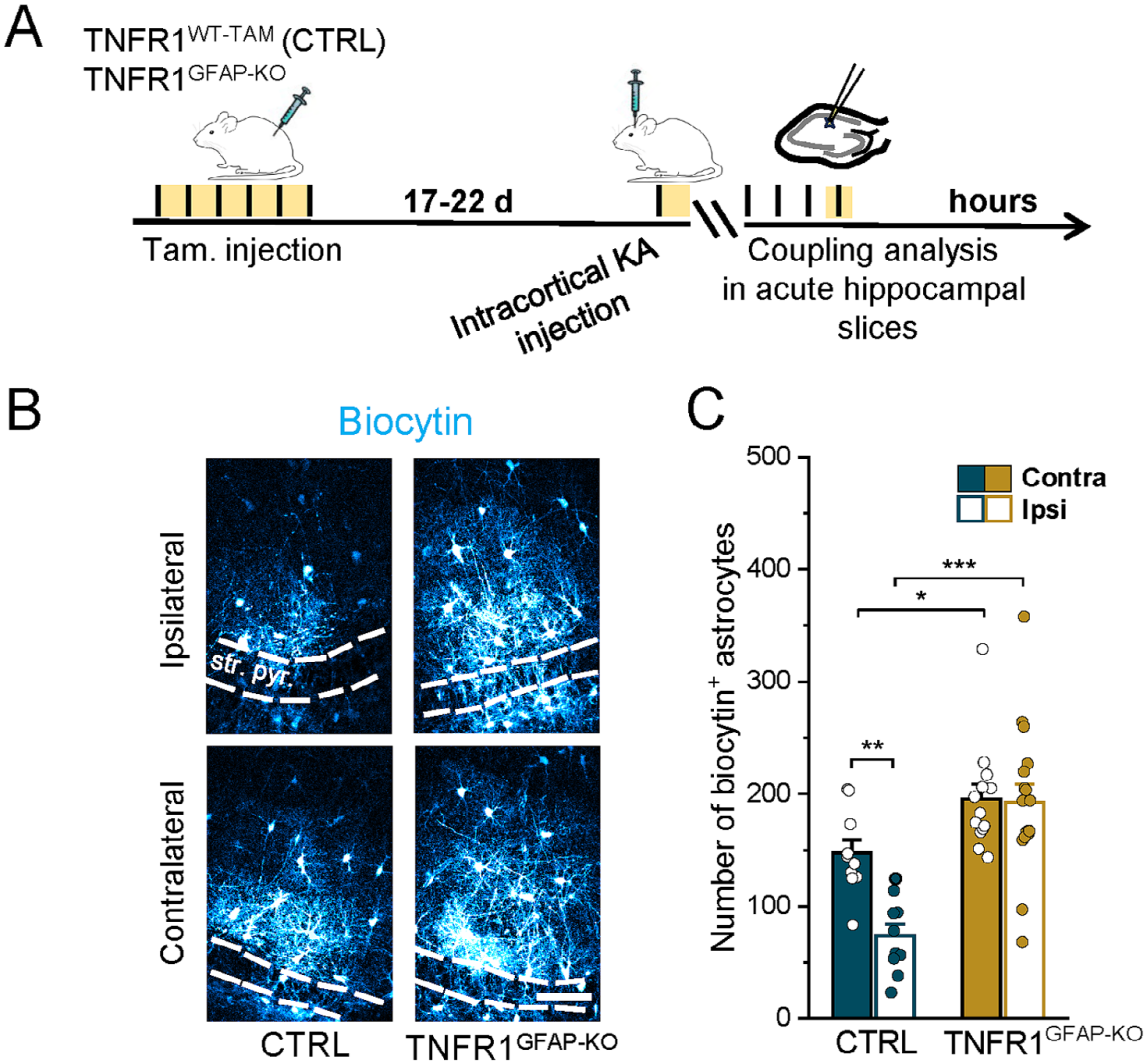
Astrocytic TNFR1 deletion prevents seizure‐induced astrocyte gap junction uncoupling. (A) Schematic of experimental procedure. TNFR1^GFAP‐KO^ and Cre‐negative TNFR1^WT‐TAM^ control mice were subjected to stereotaxic unilateral intracortical KA injections 17–21 days after TAM application. Four hours after SE induction, mice were sacrificed, and the extent of inter‐astrocytic gap junctional coupling was measured by biocytin diffusion experiments in acute coronal brain slices (Methods). (B) Representative MIPs depicting biocytin‐filled astrocytes labeled with streptavidin‐conjugated Alexa Fluor 647 in the ipsi‐ and contralateral hippocampal CA1 SR region. Scale bar: 50 μm (C) Graph summarizing the results from tracer coupling experiments. Note that KA‐induced ipsilateral reduction in astrocyte coupling was found only in control mice but not in TNFR1^GFAP‐KO^ mice (Two‐way ANOVA: Significant effect of condition (*F* = 32.531, 46, *p* < 0.001), significant effect of the site (*F* = 6.841, 46, *p* = 0.012) and significant interaction between site (contra vs. ipsi) and condition (CTRL vs. TNFR1GFAP‐KO) (*F* = 5.891, 46, *p* = 0.019)). Results of the subsequent post hoc analysis of the interaction: Ipsi versus Contra (CTRL, F1,46 = 10.68, *p* < 0.01; TNFR1GFAP‐KO, F1,46 = 0.02, *p* = 0.88); CTRL versus TNFR1GFAP‐KO (Ipsi, F1,46 = 34.94, *p* < 0.001; Contra, F1,46 = 5.09, *p* = 0.029). Data represent mean + SEM. *N* = 10 slices from 4 mice (CTRL) and 18 slices from 6 mice (KO). *p < 0.05; ***p < 0.001.

We have previously shown that disruption of astrocytic gap junctional communication is a typical feature of the sclerotic hippocampus in human and experimental TLE. Importantly, gap‐junction uncoupling and the resulting extracellular K^+^ accumulation preceded neuronal dysfunction, suggesting a causative role in TLE pathogenesis (Bedner et al. 2015). Furthermore, in the intracortical KA injection model of TLE, we could demonstrate that early astrocyte uncoupling is mediated by microglia‐released TNFα (Henning et al. 2023). Therefore, the question arose as to whether deletion of astrocytic TNFR1 prevents pathological loss of astrocytic coupling, which would provide an explanation for the attenuated course of epileptogenesis in TNFR1^GFAP‐KO^ mice. To address this question, gap junction coupling was assessed 4 h after KA injection by biocytin filling of individual hippocampal CA1 SR astrocytes in coronal brain slices (see Methods, Figure 6A). In CTRL mice, SE caused a more than 50% reduction in the number of biocytin‐positive ipsilateral astrocytes, while no change in coupling was observed in TNFR1^GFAP‐KO^ mice ipsilaterally (Figure 6B,C). Interestingly, in the contralateral hippocampus, TNFR1^GFAP‐KO^ mice displayed a higher number of coupled astrocytes than CTRL mice, suggesting that CTRL mice underwent slight TNFR1‐dependent astrocyte uncoupling also contralaterally. This assumption is further supported by the observation that contralateral coupling in CTRL differed significantly from that in non‐epileptic mice (*p* = 0.018, Figure 6C vs. Figure S2B). Under non‐epileptic conditions, however, no genotype‐dependent differences in coupling efficiency were detected (Figure S2), suggesting that in the healthy brain, astrocytic coupling is not influenced by basal TNFα levels or TNFR1 activation.

Collectively, these data show that disruption of gap junction coupling between astrocytes during early epileptogenesis in our model is dependent on astrocytic TNFR1, providing a link between TNFR1 receptor signaling and the initiation of TLE.

## 4 Discussion

The constitutively low levels of TNFα found in the healthy brain are thought to be crucial for the regulation of brain processes such as synaptic transmission and plasticity (Beattie et al. 2002; Habbas et al. 2015; Santello and Volterra 2012; Stellwagen and Malenka 2006). Importantly, these physiological functions are not exclusively mediated via neuronal TNFR1 activation but also involve TNFR1 in astrocytes. In fact, astrocyte TNFR1 signaling is a key regulator and prerequisite for astrocytic glutamate release and gliotransmission (Santello et al. 2011; Santello and Volterra 2012). Glutamatergic gliotransmission, in turn, shapes synaptic transmission and plasticity. Notably, in the hippocampus, glutamatergic gliotransmission controls pre‐synaptic release probability, post‐synaptic excitability, and firing synchronization of glutamatergic neurons (Angulo et al. 2004; de Ceglia et al. 2023; Di Castro et al. 2011; Fellin et al. 2004; Jourdain et al. 2007). In this study, we detected reduced basal EEG activity in mice devoid of astrocytic TNFR1. As EEG power reflects the number of neurons discharging simultaneously, these data provide further evidence for the importance of astrocytic TNFR1 in regulating neuronal synchronization under physiological conditions.

Numerous studies report elevated TNFα transcript and protein levels in both human and experimental epilepsy (Benson et al. 2015; De Simoni et al. 2000; Eberhard et al. 2025; Henning et al. 2023; Lachos et al. 2011; Sano et al. 2021; Vezzani et al. 2002; Yamamoto et al. 2006). While basal levels of TNFα appear to be essential for proper brain function, elevated release of the cytokine has either protective or harmful effects, depending on the magnitude and duration of the increase and the type of receptor activated (Habbas et al. 2015; Santello and Volterra 2012). Thus, TNFα‐mediated TNFR1 activation mostly promotes hyperactivity and cell death, while TNFR2 activation shows protective effects in epilepsy (Balosso et al. 2005; Patel et al. 2017; Weinberg et al. 2013). Consistent with this, mice constitutively lacking TNFR1 in all cells were found to be less susceptible to experimentally induced seizures, while mice lacking TNFR2 were more susceptible (Balosso et al. 2005; Kirkman et al. 2010; Patel et al. 2017). The antagonistic effects of TNFR1 versus TNFR2 signaling in epilepsy were also demonstrated by a study comparing the impact on KA‐induced acute seizures of selective TNFR1 activation versus TNFR1 + TNFR2 co‐activation: activation of TNFR1 alone led to exacerbation of the seizures, whereas co‐activation of the two receptors had no impact on them (Weinberg et al. 2013).

We have previously shown that TNFα release and TNFR1 activation are important events in the development of epilepsy. Notably, using the intracortical KA model of TLE, we found that reactive microglia profusely release the cytokine during epileptogenesis (Henning et al. 2023). Although TNFα release is not responsible for triggering seizures, we found that specific inhibition of soluble TNFα/TNFR1 signaling attenuated the epileptic phenotype in an epilepsy model based on selective ablation of a subpopulation of hippocampal pyramidal neurons (Eberhard et al. 2025). The cellular origin of TNFR1 signaling driving seizure activity was not investigated before. Here, we addressed whether and to what extent astrocyte TNFR1 contributes to seizure generation and epileptogenesis. Our results show that astrocyte‐selective, acute TNFR1 ablation produces significant attenuation of both acute and chronic epileptic activity, as well as of the histopathological changes induced by KA injection, providing the first experimental evidence that astrocytic TNFR1 signaling contributes to neuronal hyperexcitability and epileptogenesis.

The cellular mechanisms mediating the TNFR1 action in astrocytes are probably complex and involve several different pathological changes of these cells, among which gap‐junction uncoupling and dysregulated glutamatergic gliotransmission likely play primary roles. Moreover, it should be considered that while TNFR1‐dependent astrocytic pathology likely contributes substantially to disease progression, its prevention alone appears insufficient to fully block epileptogenesis in this multifactorial model.

Previously, we have identified in both human and experimental TLE‐HS a loss of astrocyte tracer coupling via gap‐junction channels, as well as dramatic morphological alterations (Bedner et al. 2015; Bedner and Steinhäuser 2023). Importantly, in experimental TLE, astrocytic uncoupling preceded neuronal cell death, spontaneous seizure development and sclerotic changes, suggesting a causal role in the etiology of this condition (Bedner et al. 2015). Consistently, constitutive knock‐out of astrocyte connexins was found to exacerbate chronic epileptic activity (Deshpande et al. 2020). Among the key epilepsy‐promoting consequences of a disrupted gap junctional coupling is the decreased capacity by astrocytes to buffer extracellular K^+^ during high neuronal activity (Orkand 1986) and the accumulation of Na^+^ in their cytosol. The latter phenomenon may in turn impair glutamate uptake and, by triggering reversal of the Na^+^/Ca^2+^ exchanger, increase intracellular Ca^2+^ levels, disturbing Ca^2+^‐dependent processes such as glutamatergic gliotransmission. All these astrocyte dysregulations would ultimately cause enhanced network excitability (Bedner and Steinhäuser 2023). Previously, we have shown that disruption of gap junction coupling is mediated by TNFα (Bedner et al. 2015; Henning et al. 2023). Here, we provide experimental evidence that activation of astrocytic TNFR1 is responsible for this dysfunction.

As for dysregulated glutamatergic gliotransmission, we have previously shown that, under normal conditions, picomolar levels of TNFα (most likely constitutively released from microglia) exert a permissive action on glutamate exocytosis evoked by activation of astrocyte Gq‐GPCRs (Santello et al. 2011). However, when TNFα levels increase, like in local inflammatory processes, the type of action of the cytokine on astrocyte glutamate release changes: TNFα becomes instructive, that is capable of releasing glutamate without need of GPCR activation. Moreover, this effect is concentration‐dependent, so that, at high TNFα levels, the amount of glutamate released by the cytokine exceeds the one induced by normal GPCR activation (Bezzi et al. 2001; Santello et al. 2019). This amplified release, possibly involving loops via purinergic signaling, ultimately leads to synaptic alterations including a persistently enhanced hippocampal excitatory transmission mediated by TNFR1 and NMDARs (Habbas et al. 2015; Nikolic et al. 2018; Santello et al. 2019). Hence, by dysregulating glutamatergic gliotransmission, abnormal astrocyte TNFR1 signaling may further exacerbate the proconvulsive effects that it induces via impairment of astrocyte gap‐junction coupling. Similarly, aberrant glutamatergic gliotransmission and impaired gap‐junction coupling‐mediated K^+^ buffering could promote excitotoxicity, providing a link between reduced neuronal cell loss and astrocytic TNFR1 deficiency during chronic KA‐induced epilepsy. Moreover, the reduced extent of astrogliosis in astrocytic TNFR1‐deficient mice found in this study is in line with previous results demonstrating that proinflammatory cytokines including TNFα promote reactive astrogliosis and remodeling of the astroglial cytoskeleton (Liddelow et al. 2017; Hyvärinen et al. 2019; Trindade et al. 2020). This astroglial remodeling may also contribute to granule cell dispersion by disrupting extracellular cues that maintain granule cell positioning. Future experiments will better clarify the respective contributions of these mechanisms (and possibly others) to the detrimental effects of astrocytic TNFR1 activation in epilepsy.

Concerning the temporal aspect of the noxious astrocyte TNFR1 actions, our data show a delayed onset of acute seizures after systemic KA injection in TNFR1^GFAP‐KO^ mice and a shorter total seizure duration, as well as shorter individual seizures in both the acute and chronic models, especially during the first hour after KA injection. The mechanism underlying this early protective effect of astrocyte TNFR1 deletion is unclear. This could depend on an already increased concentration of TNFα in the first stages of KA‐induced pathology, disturbing gap junction coupling and/or gliotransmission homeostasis. On the other hand, the diminished basal EEG power in TNFR1^GFAP‐KO^ mice compared to wild‐type mice is indicative of a lower level of circuit excitability, which may make TNFR1^GFAP‐KO^ mice less prone to enter seizure activity upon KA administration, and/or less apt at sustaining seizures over time once they start. In this context, abolition of TNFR1‐dependent gliotransmission in TNFR1^GFAP‐KO^ mice could also down‐regulate its neuronal receptor targets, including extra‐synaptic NMDAR, known to mediate slow excitatory inward currents that maintain seizure activity (Angulo et al. 2004; Fellin et al. 2004; Tian et al. 2005). However, in the late phase of SE, TNFα levels are clearly increased (Henning et al. 2023) and lead (in addition to sustained enhanced glutamate transmission) to loss of gap junction coupling efficiency and other astrocytic changes, which can further promote neuronal hyperactivity and trigger neurodegeneration, thus initiating epileptogenesis.

During the latent and chronic phases in the TLE‐HS model, TNFR1^GFAP‐KO^ mice displayed SGS frequency similar to control mice, but lower spike frequency and EEG power, indicating reduced interictal spiking activity. This activity represents an electrographic marker of the epileptic brain, but the mechanisms underlying its development and relationship to ictal activity are still uncertain (de Curtis et al. 2012; de Curtis and Avanzini 2001). Interictal discharges are produced by synchronous firing of clusters of hyperexcitable neurons different from those responsible for seizure generation (de Curtis and Avanzini 2001). At the cellular level, they are characterized by abnormally prolonged neuronal depolarizations with superimposed action potentials, the so‐called paroxysmal depolarization shifts (PDSs). Glutamate released by astrocytes can induce PDSs in neurons (Kang et al. 2005; Tian et al. 2005); thus, a TNFα‐mediated increase in gliotransmission may underlie increased interictal activity, an effect that is suppressed in TNFR1^GFAP‐KO^ mice. On the other hand, in KA‐treated CTRL mice, astrocytic uncoupling is known to persist and even exacerbate over months (Bedner et al. 2015), suggesting that loss of coupling efficiency may contribute not only to the initiation but also to the progression of epilepsy possibly with different sustaining mechanisms. The consequent disturbance of K^+^ and glutamate buffering promotes a hyperexcitable environment that favors emergence of PDSs and thus interictal activity. Consistent with this notion, mice with constitutive deletion of astrocytic connexins display increased interictal activity (Deshpande et al. 2020).

Further experiments are needed to fully clarify the mechanism by which astrocyte TNFR1 influences epileptogenesis. Importantly, in our *GFAP* ^*creERT2*^ *TNFR1* ^*flox/flox*^ *tdTomato* ^*lsl/lsl*^ mice, the efficiency of Cre‐induced recombination was 50%–60%, implying that a substantial number of astrocytes remained sensitive to the effects of TNFR1 signaling triggered by KA administration. Thus, an even more pronounced seizure‐suppressing phenotype can be expected in mice displaying higher recombination efficiency in hippocampal CA1 astrocytes (Bohmbach et al. 2022).

## 5 Conclusions

The neuroinflammatory cytokine TNFα has previously been implicated in the pathophysiology of epilepsy, but the dichotomy of TNFR1 versus TNFR2 activation, the diversity of TNFR‐expressing cell types, and the complexity of TNFRs downstream signaling have obscured the precise involvement of the cytokine in the genesis of the disease. The present study provides novel insights into the role of TNFα in epilepsy and identifies astrocyte TNFR1 signaling as a key mediator of epileptogenesis. We demonstrate that inducible, astrocyte‐specific deletion of TNFR1 mitigates the initiation of epilepsy by dampening and delaying acute seizure activity in two different models of epilepsy, providing cross‐model evidence for its involvement in the generation of hyperexcitability. In addition, it attenuated chronic epileptiform activity and the characteristic histopathological changes in a well‐established mouse model of TLE‐HS. Mechanistically, astrocyte‐specific TNFR1 deletion not only attenuated basal spectral EEG power under physiological conditions but also preserved astrocytic gap junction coupling during intracortical KA‐induced SE. The spatial and temporal scale at which both phenomena contribute to the observed reduction in the acute and chronic epileptiform activity, as well as their precise molecular mechanisms, remain to be elucidated. Moreover, despite convergent observations, our results in the systemic and intracortical KA administration models need to be extrapolated with some caution with respect to human TLE in view of significant intermodel differences, including short‐ and long‐term histopathological, ictal and behavioral manifestations (Rusina et al. 2021; Xing et al. 2026). Nevertheless, our findings implicate aberrant astrocyte TNFR1 signaling in the broader context of epileptogenesis and seizure susceptibility following an epileptogenic brain insult. Consequently, they identify astrocyte TNFR1 as a novel potential drug target in focal epilepsy, including forms that are resistant to current therapies.

## Author Contributions

Lukas Henning designed, performed and analyzed in vivo experiments in the intracortical KA injection model; performed and quantified the coupling and IHC analysis, prepared the figures and wrote the manuscript’ sections related to them, and contributed to the overall manuscript writing; Ioannis Zalachoras designed, performed and analyzed in vivo experiments in the systemic KA injection model, prepared the cell suspensions for FACS‐isolation, performed genomic PCR experiments, prepared the figures and wrote the manuscript’ sections related to them, and contributed to the overall manuscript writing; Giovanni Carriero set up systemic KA experiments and spectral EEG data analysis; Peter Bedner co‐designed the study, performed and analyzed in vivo experiments in the intracortical KA injection model, participated to analysis and figures preparation and wrote the manuscript; Christian Steinhäuser and Andrea Volterra co‐designed the study, supervised the experiments in the system and intracortical KA injection model, defined their strategy and, together with the other authors, designed the different components of the study, participated to analysis and figures preparation and wrote the manuscript.

## Funding

This work was supported for the Volterra lab. by Innosuisse Innovation project no. 48812.1 IP‐LS, by Swiss National Science Foundation (SNSF) grant numbers 31003A‐173124 and 31003B‐201276, and the Wyss Center for Bio and Neuroengineering, Geneva; for the Bonn group support was from Deutsche Forschungsgemeinschaft (grant number STE 552/9 to C.S.).

## Conflicts of Interest

Andrea Volterra is co‐founder of the company Aleos Bio Ltd. The other authors declare no conflicts of interest.

## Supporting information


**Figure S1:** The two control groups TNFR1WT‐TAM and TNFR1GFAP‐WT were not different in any of the seizure‐related parameters analyzed upon systemic KA injection (see Figure 2).
**Figure S2:** Basal astrocyte gap junctional coupling is not affected by astrocyte‐specific deletion of TNFR1.

## Data Availability

The data that support the findings of this study are available from the corresponding author upon reasonable request.
