# Supplementary material for: Deletion of Astrocytic TNFR1 Attenuates Hyperexcitability and Initiation of Epileptogenesis
Source: Glia. 2026 Jul 20;74(9):e70205. doi: 10.1002/glia.70205 (PMC13382600; doi:10.1002/glia.70205)
Supplement: Supplementary file 1 — Figure S1: The two control groups TNFR1WT‐TAM and TNFR1GFAP‐WT were not different in any of the seizure‐related parameters analyzed upon systemic KA injection (see Figure 2). Figure S2: Basal astrocyte gap junctional coupling is not affected by astrocyte‐specific deletion of TNFR1. [file GLIA-74-0-s001.pdf]

Supplementary information

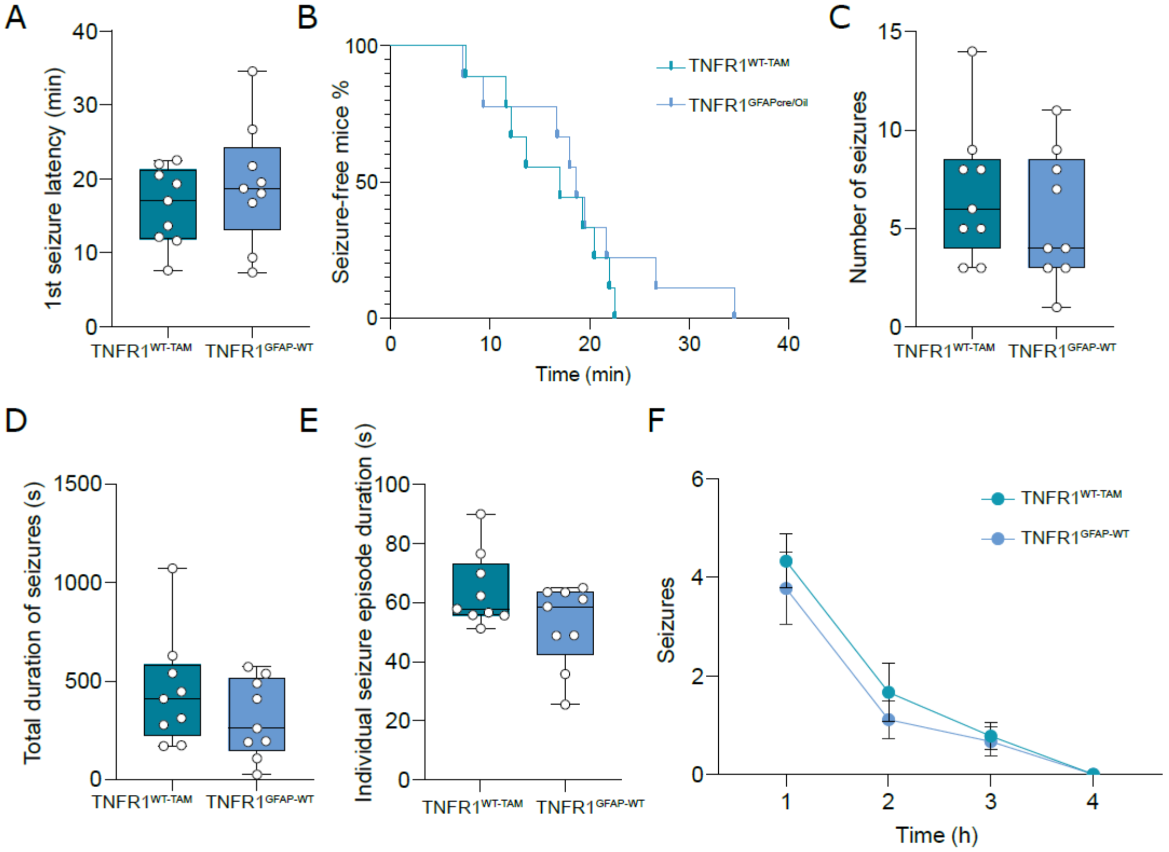

Supplementary Figure 1. The two control groups $TNFR1^{WT\text{-}TAM}$ and $TNFR1^{GFAP\text{-}WT}$ were not different in any of the seizure-related parameters analyzed upon systemic KA injection (see Fig. 2).

List of parameters tested with respective statistical comparison between $TNFR1^{WT\text{-}TAM}$ and $TNFR1^{GFAP\text{-}WT}$ mice **A.** 1st seizure latency following KA treatment (two-tailed Welch`s t-test, $t_{(13.58)} = 0.8956$, $p = 0.386$). **B.** Percentage of seizure-free mice over time (Mantel-Cox test, Chi-square = 0.649, $p = 0.420$). **C.** Number of seizures: (two-tailed Welch`s t-test, $t_{(15.97)} = 0.7650$, $p = 0.455$). **D.** Total duration of seizures: (two-tailed Welch`s t-test, $t_{(14.38)} = 1.203$, $p = 0.248$; $p = 9$). **E.** Average duration of individual seizures: (two-tailed Welch`s t-test, $t_{(15.83)} = 1.869$, $p = 0.080$). **F.** The number of seizures over time: Two-way ANOVA: Time x Group interaction ($F_{(3, 48)} = 0.2879$, $p = 0.834$), Group effect (($F_{(1,16)} = 0.5852$, $p = 0.4554$), Time effect ($F_{(2.225, 35.61)} = 42.22$, $p < 0.001$).

Data in panels A, C, D, E, are shown as boxplots with median and 25-75 percentiles, with whiskers indicating minimum-maximum values. Panel B shows the proportion of seizure-free mice at each time point for both groups. In panel F data are shown as mean ± SEM. N = 9 mice per each group, $TNFR1^{WT\text{-}TAM}$ and $TNFR1^{GFAP\text{-}WT}$.

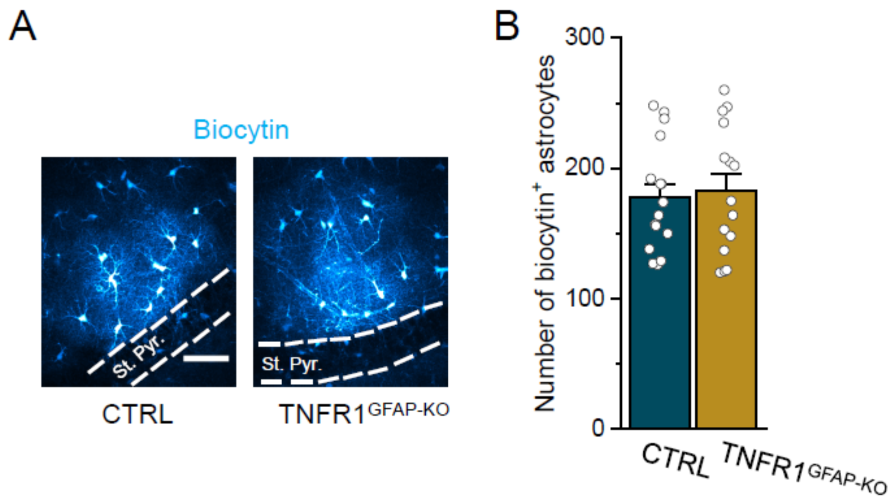

Supplementary Figure 2: Basal astrocyte gap junctional coupling is not affected by astrocyte-specific deletion of TNFR1.

Experimental plan as in Fig. 6 but without KA treatment. The extent of inter-astrocytic gap junctional coupling was measured by biocytin diffusion experiments in acute coronal brain slices from TNFR1$^{GFAP\text{-}KO}$ and TNFR1$^{WT\text{-}TAM}$ control mice 17-21 days after TAM injection.

A. Representative MIPs depicting biocytin-filled astrocytes labeled with streptavidin-conjugated Alexa Fluor® 647 in the hippocampal CA1 *str. rad.* bordering the pyramidal cell layer *(St. Pyr.)* in the two groups of mice. Scale bar: 50 µm

B. Graph summarizing the results from tracer coupling experiments. Note that the extent of biocytin diffusion was not different between genotypes.

Data represent mean ± SEM. N = 14 slices from 3 mice (CTRL) and 13 slices from 3 mice (TNFR1$^{GFAP\text{-}KO}$). Welch`s two-tailed t-test ($t_{23.7} = 0.36$, $p = 0.72$) was used.
